# Novel utilization of the seaweed-based deoxy sugars rhamnose and fucose by engineered *Corynebacterium glutamicum*

**DOI:** 10.1186/s12934-026-03035-5

**Published:** 2026-06-11

**Authors:** Ida Marie Stephansen, Luciana Fernandes Brito, Oliver Saur Auran, Synne Saether Lande, Fernando Pérez-García

**Affiliations:** Department of Biotechnology and Food Science, Faculty of Natural Sciences, NTNU, Trondheim, Norway

**Keywords:** l-rhamnose, l-fucose, *Corynebacterium glutamicum*, Seaweed hydrolysate, Deoxy sugars, Lactaldehyde

## Abstract

Seaweed represents a potential sustainable carbon source for industrial biotechnology, yet the workhorse bacterium *Corynebacterium glutamicum* cannot naturally metabolize the deoxy sugars l-rhamnose and l-fucose, abundant in green and brown seaweed, respectively. Expanding its substrate range is crucial for sustainable bioprocessing, by enabling utilization of the available biomass. In this study, we engineered *C. glutamicum* to utilize l-rhamnose and l-fucose by introducing the *Escherichia coli* operons *rhaBADM* and *fucIKUA*, enabling growth on these sugars as sole carbon sources. To enhance growth, we evaluated various transport systems and identified the non-native fucose permease (FucP) as the most efficient for l-rhamnose uptake, and the native *myo*-inositol transporter 2 (IolT2) as optimal for l-fucose uptake. During l-rhamnose and l-fucose utilization, l-lactaldehyde is formed as a byproduct. We demonstrate that the native acetaldehyde dehydrogenase encoded by *ald* also exhibits lactaldehyde dehydrogenase activity, and that its overexpression enhances l-lactaldehyde utilization. Finally, cultivation on green and brown seaweed hydrolysates enabled the engineered strains to achieve increased biomass formation through consumption of the targeted deoxy sugars. This study expands the substrate spectrum of *C. glutamicum* through pathway engineering, transport optimization, and functional identification of a native lactaldehyde dehydrogenase. Growth and l-lysine production on seaweed hydrolysates highlights the potential of this approach for sustainable marine biomass valorization and bioproduction of value-added compounds.

**Supplementary Information:**

The online version contains supplementary material available at 10.1186/s12934-026-03035-5.

## Introduction

The global population exceeds 8 billion and increases by about 70 million people annually, intensifying demand for sustainable feedstocks [1, 2]. Additionally, there is increasing interest in sustainable carbon sources for bioprocesses that do not compete with food and feed industries [3, 4]. In industrial biotechnology, d-glucose from crops is widely used for microbial factories, which raises ethical concerns about diverting food crops for industrial energy production. In this context, alternative feedstocks are being investigated as carbon sources in biotechnology, such as CO_2_, methanol, and biomass feedstocks like seaweed [5–11].

Seaweeds, or macroalgae, offer a compelling alternative to terrestrial crops due to their rapid growth rates and minimal resource demands; they do not require arable land, freshwater, or fertilizers [12]. In 2020, global seaweed production exceeded 35 million tonnes, mainly cultivated in Indonesia, China, and the Philippines, with great potential for the seaweed industry in Norway [13–15]. Seaweed biomass contains a variety of fermentable carbohydrates such as d-glucose, d-mannitol, alginate, and d-xylose, which are suitable substrates for various microorganisms [16]. These carbohydrates typically account for 30–60% of the seaweed dry weight, though the carbohydrate composition of seaweed varies significantly depending on species, harvest time, and geographic location [17–19]. The seaweed industry has mainly focused on the extraction of compounds for use as thickeners, and these industries have side streams containing valuable carbohydrates that should be exploited [20, 21]. Several studies have analyzed the leftovers of seaweeds after agar, alginates, and carrageenan were extracted, and found that 44–47% of the carbohydrates remained, leaving a significant potential for industrial biotechnology to be more sustainable [22, 23].

Recent biotechnological advances have demonstrated the utilization of seaweed-derived carbohydrates, such as alginate, laminarin, mannitol, galactose, and xylose, as a carbon source for bacteria and yeast, coupled with the production of value-added compounds, such as polyhydroxyalkanoates, l-lysine, riboflavin, l-lactic acid, biomethane, and bioplastics [24–32].

**Fig. 1:**
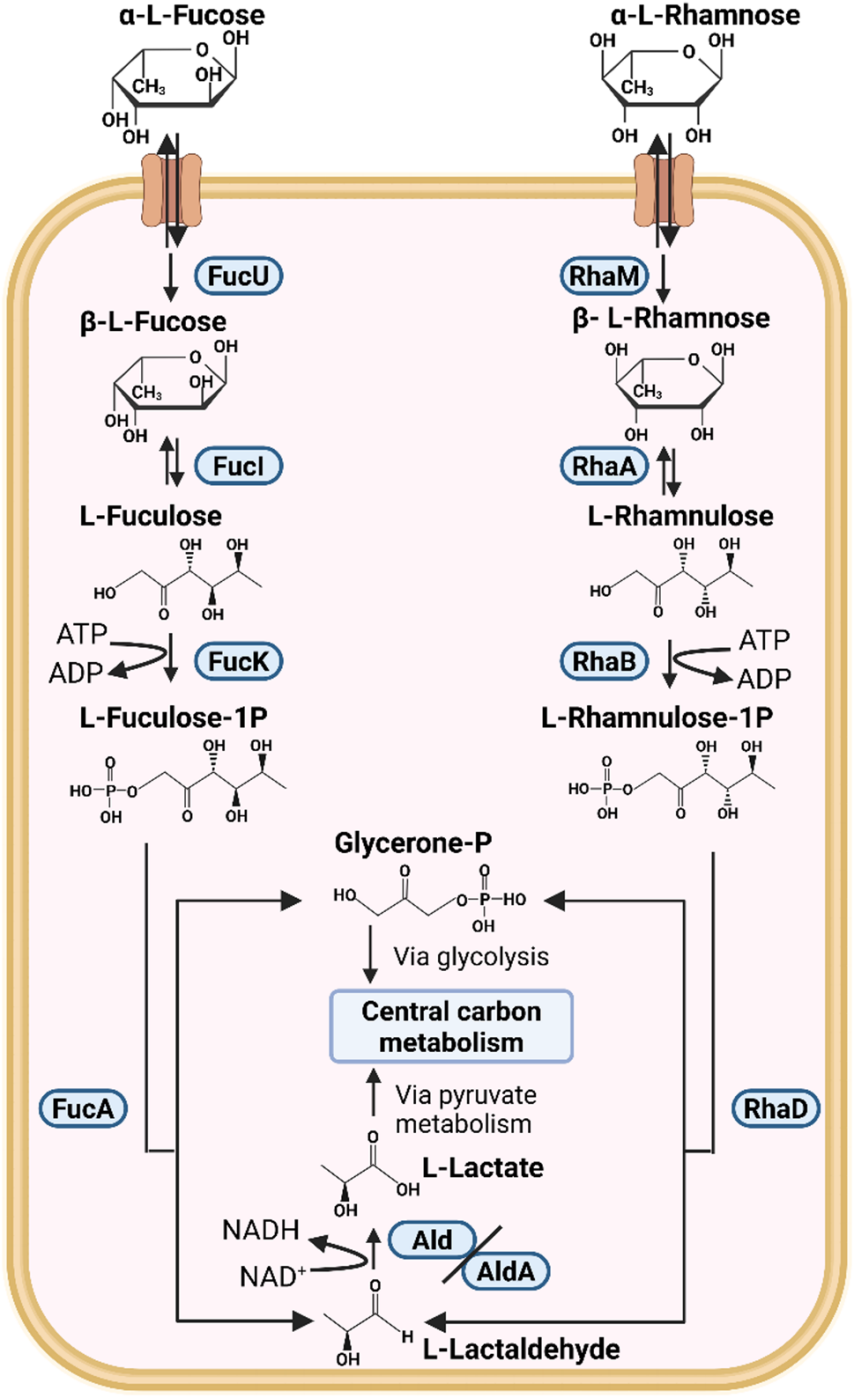
Schematic representation of the metabolic pathways for the utilization of l-rhamnose and l-fucose. FucU: l-fucose mutarotase; FucI: l‑fucose isomerase; FucK: l‑fuculokinase; FucA: l‑fuculose‑1‑phosphate aldolase; RhaM: l-rhamnose mutarotase; RhaA: l‑rhamnose isomerase; RhaB: l‑rhamnulokinase; RhaD: l‑rhamnulose‑1‑phosphate aldolase; AldA: lactaldehyde dehydrogenase; Ald: acetaldehyde dehydrogenase/lactaldehyde dehydrogenase. Created in BioRender. Stephansen I. (2026) https://BioRender.com/llumw9o

Based on this information, seaweed is a promising biomass feedstock for the sustainable production of value-added products. The green seaweed *Ulva lactuca* and the brown seaweed *Saccharina latissima* contain, among other sugars, the deoxy sugars l-rhamnose and l-fucose, respectively [33]. l-Rhamnose and l-fucose are deoxyhexoses found in several organisms, such as bacteria, plants, and seaweed [34, 35]. They are unusual sugars lacking the hydroxyl group at the C6 position and occur naturally in l-configuration, unlike the other natural sugars, which are d-configured [36]. l-Rhamnose and l-fucose are monomers composing the polysaccharides ulvan and rhamnan, and fucoidan, respectively, which are sulfated polymers typically constituting components of the the cell wall of the seaweeds [37, 38]. Green seaweed contains 8–29% ulvan, of which l-rhamnose accounts for 16.8–45% of the dry weight [37]. Fucoidan is found in brown seaweed and consists mainly of l-fucose and sulfate, and is commonly found in a range of 1–13% of the dry weight [38–41]. In *S. latissima* specifically, l-fucose was reported to compose 0.82–1.5% of the dry weight [17]. l-Rhamnose and l-fucose are naturally metabolized in *E. coli* and *Clostridium phytofermentans*, for instance [42, 43]. However, these deoxy sugars are non-native carbon sources for *C. glutamicum*. *C. glutamicum* is an industrially important workhorse used in the million-ton-scale industry of the production of amino acids l-glutamate and l-lysine, used as food and feed additives [44–46]. This bacterium has been extensively engineered to acquire new capabilities, including the utilization of various non-native carbon sources and the production of diverse value-added compounds [47, 48]. In industrial biotechnology, *C. glutamicum* is often cultivated using d-glucose derived from crops as feedstock, which reinforces the appeal of exploring novel, sustainable carbon sources for production [8, 49–51]. Moreover, *C. glutamicum* lacks a complex carbon catabolite repression system, allowing it to utilize several sugars simultaneously, in contrast to *E. coli* [52]. The substrate spectrum of *C. glutamicum* has previously been expanded with several seaweed carbohydrates, such as d-galactose and d-xylose, found in red seaweed [30, 53], and mannitol, a sugar alcohol found in brown seaweed [29]. Targeting l-rhamnose and l-fucose expands the fraction of fermentable carbon in seaweed hydrolysates accessible to *C. glutamicum*, advancing marine biomass valorization [37, 41]. In this study, we aimed to enable *C. glutamicum* to utilize the two non-native seaweed-derived deoxy sugars, l-rhamnose and l-fucose, by using *E. coli*-derived operons. We investigated various transport proteins to optimize the import and utilization of these sugars. Furthermore, we identified a functional lactaldehyde dehydrogenase, encoded by *ald*, in *C. glutamicum* that enables the utilization of l-lactaldehyde, a byproduct of the l-rhamnose and l-fucose metabolism, as a carbon source. Finally, we demonstrated growth and l-lysine production of our engineered *C. glutamicum* strains using seaweed hydrolysates from *S. latissima* and *U. lactuca* as the sole carbon source (Fig. 1).

## Methods

### Bacterial strains, plasmids, and growth conditions

The plasmids and bacterial strains used in this work are listed in Tables 1 and 2, respectively. Unless stated otherwise, all chemicals and consumables used in this study were purchased from Sigma-Aldrich. The incubator used in this study was a New Brunswick Innova^®^ 42 (Eppendorf). *E. coli* DH5α, routinely used as a cloning host, was cultivated in Lysogeny Broth (LB; 10 g L^− 1^ tryptone, 5 g L^− 1^ yeast extract, and 5 g L^− 1^ NaCl) at 225 rpm or on LB agar plates at 37 °C, while *C. glutamicum*, used as an expression host, was grown in 2TY (16 g L^− 1^ tryptone, 10 g L^− 1^ yeast extract, and 5 g L^− 1^ NaCl) for pre-cultures at 225 rpm and 30 °C. Main cultures were grown in baffled shake flasks, either 250 mL or 500 mL, with a 10% cultivation volume, at 150 rpm shaking and 30 °C. Cultivations were also performed in a BioLector Pro (m2p Labs) microbioreactor, with 1 mL of culture at 30 °C and 1100 rpm, using a 48-well FlowerPlate and gas-permeable sealing foil (Beckman Coulter). Growth experiments performed using the BioLector system were monitored by measuring the backscatter signal at 620 nm. This provides values in arbitrary units (a.u.) that correlate with the cell density of the culture. Minimal medium (CGXII) supplemented with biotin (0.2 mg L^− 1^) and 1 mL L^− 1^ trace metals solution was prepared as described elsewhere [54]. The CGXII salt solution consists of (NH_4_)_2_SO_4_ (10 g L^− 1^), KH_2_PO_4_ (1 g L^− 1^), K_2_HPO_4_ (1 g L^− 1^), urea (5 g L^− 1^), MOPS (42 g L^− 1^), 1 mL of Mg-stock (MgSO_4_·7H_2_0, 250 g L^− 1^), and 1 mL of Ca-stock (CaCl_2_·2H_2_0, 13.25 g L^− 1^). The trace metals solution consisted of FeSO_4_·7H_2_0 (16.4 g L^− 1^), MnSO_4_·H_2_0 (10 g L^− 1^), ZnSO_4_·7H_2_0 (1 g L^− 1^), CuSO_4_·5H_2_0 (0.31 g L^− 1^), and NiCl_2_·6H_2_0 (0.02 g L^− 1^), and was pH adjusted to 1 with HCl. The main cultures were inoculated to an initial optical density at 600 nm (OD_600_) of approximately 1. Optical densities were measured via the spectrophotometer Biochrom Ultrospec 7500 (Fisher Scientific). The cells were washed with CGXII solution once before inoculating the minimal medium. Cell dry biomass (g L^− 1^) was estimated as 0.343·x (OD_600__final - OD_600__initial), where 0.343 is the specific lab correlation factor for *C. glutamicum* [29]. Biomass yield was calculated by dividing the grams of biomass by the grams of carbon source utilized in the cultivations. As the carbon source, the sugars l-rhamnose, l-fucose, d-glucose, and green and brown seaweed hydrolysate were used. When necessary, 1 mM isopropyl β-d-thiogalactopyranoside (IPTG), 5 µg mL^− 1^ tetracycline, and/or 25 µg mL^− 1^ kanamycin were supplemented to the medium. All media used in this study were autoclaved at 121 °C for 20 min, except for trace metals solution, antibiotics, biotin, and IPTG, which were filter-sterilized using 0.22 μm pore-size filters.

**Table 1:** List of plasmids used in this work

Plasmid name	Description	Source
pECXT99a	Tet^r^, *C. glutamicum/E. coli* shuttle plasmid (*P*_*trc*_ *lacI*, pGA1 oriVCg)	Kirchner and Tauch [55]
pVWEx1	Kan^r^, *C. glutamicum/E. coli* shuttle plasmid (*P*_*trc*_ *lacI*, pBL1 oriV_Cg_)	Peters-Wendisch et al. [56]
pSH1	Kan^r^, *C. glutamicum/E. coli* expression shuttle plasmid, P_*tuf*_, pHM519 oriV_*Cg*_	Henke et al. [57]
pVWEx1-*rhaBADM*	Kan^r^, pVWEx1 overexpressing *rhaBADM* genes from *E. coli* MG1655	This work
pVWEx1-*fucIKUA*	Kan^r^, pVWEx1 overexpressing *fucIKUA* genes from *E. coli* MG1655	This work
pECXT99a-*fucP*	Tet^r^, pECXT99a overexpressing *fucP* gene from *E. coli* MG1655	This work
pECXT99a-*rhaT*	Tet^r^, pECXT99a overexpressing *rhaT* gene from *E. coli* MG1655	This work
pECXT99a-*iolT1*	Tet^r^, pECXT99a overexpressing *iolT1* gene from *C. glutamicum* ATCC13032	Brito et al. [30]
pECXT99a-*iolT2*	Tet^r^, pECXT99a overexpressing *iolT2* gene from *C. glutamicum* ATCC13032	Brito et al. [30]
pSH1-*fucIKUA*	Kan^r^, pSH1 overexpressing *fucIKUA* genes from *E. coli* MG1655	This work
pSH1-*rhaBADM*	Kan^r^, pSH1 overexpressing *rhaBADM* genes from *E. coli* MG1655	This work
pSH1-*rhaBADM-rhaT*	Kan^r^, pSH1 overexpressing *rhaBADM and rhaT* genes from *E. coli* MG1655	This work
pSH1-*rhaBADM-fucP*	Kan^r^, pSH1 overexpressing *rhaBADM and fucP* genes from *E. coli* MG1655	This work
pSH1-*rhaBADM-iolT2*	Kan^r^, pSH1 overexpressing *rhaBADM* genes from *E. coli* MG1655 and *iolT2* gene from *C. glutamicum* ATCC13032	This work
pSH1-*fucIKUA-fucP*	Kan^r^, pSH1 overexpressing *fucIKUA and* *fucP* genes from *E. coli* MG1655	This work
pSH1-*fucIKUA-iolT2*	Kan^r^, pSH1 overexpressing *fucIKUA* genes from *E. coli* MG1655 and *iolT2* gene from *C. glutamicum* ATCC13032	This work
pVWEx1-*rhaBADM-fucP*	Kan^r^, pVWEx1 overexpressing *rhaBADM and fucP* genes from *E. coli* MG1655	This work
pVWEx1-*fucIKUA-iolT2*	Kan^r^, pVWEx1 overexpressing *fucIKUA* genes from *E. coli* MG1655 and *iolT2* gene from *C. glutamicum* ATCC13032	This work
pECXT99a-*aldA*	Tet^r^, pECXT99a overexpressing *aldA* gene from *E. coli* MG1655	This work
pECXT99a-*ald*	Tet^r^, pECXT99a overexpressing *ald* gene from *C. glutamicum* ATCC13032	This work

Kan^r^: Kanamycin resistance, Tet^r^: Tetracycline resistance

**Table 2:** List of strains used in this work

Strain name	Description	Source
*Escherichia coli* DH5α	DH5α Strain: Δ*lacU*169 (φ80*lacZ* ΔM15), *supE*44, *hsdR*17, *recA*1, *endA*1, *gyrA*96, *thi*-1, *relA*1	Hanahan [58]
*Corynebacterium glutamicum*	Wild-type strain ATCC13032, auxotrophic for biotin	Abe et al. [59]
GSLA	*C. glutamicum* ATCC13032 with the following modifications: *Δpck*, *pyc*^*P458S*^, *hom*^*V59A*^, 2 copies of *lysC*^*T311I*^, 2 copies of *asd*, 2 copies of *dapA*, 2 copies of *dapB*, 2 copies of *ddh*, 2 copies of *lysA*, 2 copies of *lysE*, in-frame deletion of prophages CGP1 (cg1507-cg1524), CGP2 (cg1746-cg1752), CGP3 (cg1890-cg2071), *sugR* (cg2115), *ldhA*(cg3219), *snaA*(cg1722)	Pérez-García et al. [60]
WT*Δald*	*C. glutamicum* wild-type strain ATCC13032 with the deletion of the acetaldehyde dehydrogenase gene (*ald*)	Lessmeiser et al. [61]
WT*ΔfadH*	*C. glutamicum* wild-type strain ATCC13032 with the deletion of the formaldehyde dehydrogenase gene (*fadH*)	Lessmeiser et al. [61]
WT*ΔaldΔfadH*	*C. glutamicum* wild-type strain ATCC13032 with the deletion of the *ald* and *fadH* genes	Lessmeiser et al. [61]
CgCon1	*C. glutamicum*(pVWEx1)	This work
CgCon2	*C. glutamicum*(pSH1)	This work
CgCon3	*C. glutamicum*(pECXT99a)	This work
CgCon4	*C. glutamicum*(pSH1)(pECXT99a)	This work
CgRha1	*C. glutamicum*(pVWEx1-*rhaBADM*)	This work
CgRha1C	*C. glutamicum*(pVWEx1-*rhaBADM*)(pECXT99a)	This work
CgRha2	*C. glutamicum*(pVWEx1-*rhaBADM*)(pECXT99a-*rhaT*)	This work
CgRha3	*C. glutamicum*(pVWEx1-*rhaBADM*)(pECXT99a-*fucP*)	This work
CgRha4	*C. glutamicum*(pVWEx1-*rhaBADM)*(pECXT99a-*iolT1*)	This work
CgRha5	*C. glutamicum*(pVWEx1-*rhaBADM*)(pECXT99a-*iolT2*)	This work
CgRha6	*C. glutamicum*(pVWEx1-*rhaBADM-fucP*)	This work
CgRha7	*C. glutamicum*(pSH1-*rhaBADM-fucP*)	This work
CgRha8	*C. glutamicum(*pSH1*-rhaBADM-fucP)(*pECXT99a)	This work
CgRha9	*C. glutamicum(*pSH1*-rhaBADM-fucP)(*pECXT99a-*ald*)	This work
CgFuc1	*C. glutamicum*(pVWEx1-*fucIKUA*)	This work
CgFuc1C	*C. glutamicum*(pVWEx1-*fucIKUA*)(pECXT99a)	This work
CgFuc2	*C. glutamicum*(pVWEx1-*fucIKUA*)(pECXT99a-*rhaT*)	This work
CgFuc3	*C. glutamicum*(pVWEx1-*fucIKUA*)(pECXT99a-*fucP*)	This work
CgFuc4	*C. glutamicum*(pVWEx1-*fucIKUA*)(pECXT99a-*iolT1*)	This work
CgFuc5	*C. glutamicum*(pVWEx1-*fucIKUA*)(pECXT99a-*iolT2*)	This work
CgFuc6	*C. glutamicum*(pVWEx1-*fucIKUA-iolT2*)	This work
CgFuc7	*C. glutamicum*(pSH1-*fucIKUA-iolT2*)	This work
CgFuc8	*C. glutamicum*(pSH1-*fucIKUA-iolT2*)(pECXT99a*)*	This work
CgFuc9	*C. glutamicum*(pSH1-*fucIKUA-iolT2*)(pECXT99a-*ald)*	This work
CgAld	*C. glutamicum* (pECXT99a-*ald)*	This work
CgAldA	*C. glutamicum* (pECXT99a-*aldA*)	This work
CgLysRha	*C. glutamicum* GSL-A (pSH1-*rhaBADM-fucP*)(pECXT99a-*ald)*	This work
CgLysFuc	*C. glutamicum* GSL-A (pSH1-*fucIKUA-iolT2*)(pECXT99a-*ald)*	This work

### Molecular genetic techniques and plasmid construction

Established molecular genetics techniques were employed as described elsewhere [62]. CloneAmp™ HiFi PCR Premix (Takara Bio Inc.) was used for the amplification of specific genes. Primers are presented in the supplementary material (Table S1). The genes *rhaBADM* (NCBI GeneIDs: 948399, 948400, 948401, 948402), *rhaT* (NCBI GeneID: 948398), *fucIKU* (NCBI GeneIDs: 946195, 946022, 945842), *fucA* (NCBI GeneID: 947282), *fucP* (NCBI GeneID: 947487), and *aldA* (NCBI GeneID: 945672) were amplified from the genomic DNA (gDNA) of *E. coli* MG1655 (Table S2). The genes *iolT1* (NCBI GeneID: 1021246), *iolT2* (NCBI GeneID: 1021001), and *ald* (NCBI GeneID: 1182348, also named *exaC*) were amplified from gDNA of *C. glutamicum* ATCC13032 (Table S2). The plasmids pVWEx1 [56], pECXT99a [55], and pSH1 [57] were linearized using BamHI (NEB). Gibson assembly was used to assemble the linearized plasmids and the amplified PCR products, following the one-step isothermal DNA assembly protocol, with incubation at 50 °C for 1 h [63]. *E. coli* was transformed via heat shock [62]. For colony PCR, GoTaq^®^ DNA polymerase (Promega) was used following the manufacturer’s instructions. The correctness of plasmid sequences was verified with Sanger sequencing (Eurofins). Transformation of *C. glutamicum* was performed with electroporation using Elepo21 (Nepa gene), in a cooled cuvette with 150 µL competent cells and 300–1000 ng plasmid. The settings used were: 1 poring pulse at 2 kV, 3.5 ms pulse length, 50 ms pulse interval, + polarity, and 3 transfer pulses with 0.2 kV, 50 ms pulse length, 50 ms pulse interval, and +/- polarity. Electroporation was followed by a heat shock at 46 °C for 5 min 45 s and recovery in 2TY for 1 h at 30 °C, before plating on selective agar and incubation overnight at 30 °C [54].

### Preparation of seaweed hydrolysates

Two seaweed species, *S. latissima* and *U. lactuca*, were used for the preparation of brown seaweed hydrolysate (BSH) and green seaweed hydrolysate (GSH), respectively. *S. latissima* was provided by Seaweed Solutions AS, and *U. lactuca* was provided by Statsnail AS, both companies located in Norway. Frozen seaweed was thawed overnight at 4 °C before it was milled in a blender. Hot water (~70 °C) was added to the chopped seaweed in a 1:1 ratio, assuming 10% water in the wet weight of the biomass. One percent of sulfuric acid (99.9%) was added to the mixture. Subsequently, the mixture was incubated with stirring in a water bath at 70 °C for 2 h before being autoclaved for 1 h at 121 °C. Next, the pH was adjusted to 5 using 4 M KOH. For enzymatic digestion of the polymers, 1% (v/v) Cellulase Enzyme Blend, Cellic CTect2, was added, and the hydrolysate was incubated overnight at 50 °C and 100 rpm. The hydrolysates were centrifuged (Sorvall Lynx 6000, Thermo Scientific) at 20,000 rpm for 30 min at 4 °C. Then, the supernatant was collected, pH-adjusted to 7 using 5 M KOH, followed by autoclaving at 121 °C for 20 min. If fragments were observed in the hydrolysate, the hydrolysate was filtered using a 0.2 μm filter.

### HPLC analyses

High-performance liquid chromatography (HPLC) (Waters Alliance e2695 Separations Module) was used for the quantification of extracellular carbohydrates, l-lysine, 5-hydroxymethylfurfural (5HMF), and furfural in seaweed hydrolysates and cell culture supernatants. To collect supernatants, 1 mL of culture was transferred into a microcentrifuge tube and centrifuged at 13,300 rpm (VWR^®^ MicRoStaR 17R) for 15–30 min to remove cells. The supernatant was then transferred to a new tube and frozen at -20 °C until use. Supernatants for quantification of carbohydrates were diluted 1:20 in Milli-Q water. For carbohydrates analysis, furfural and 5HMF were quantified using an Aminex HPX-87 H column (300 mm x 7.8 mm, Bio-Rad) at 60 °C, with detection by a refractive index detector (2414 RI Detector, Waters). As the mobile phase, 5 mM sulfuric acid was used, running isocratically at a 0.6 mL min^− 1^ flow rate. For the quantification of l-lysine, the supernatants were derivatized with 9-fluorenylmethyl chloroformate (FMOC-CI), and the method was carried out using a gradient flow as described by Brito et al. (2021) [64], with 50 mM Na-acetate (pH 4.2) and acetonitrile as the mobile phases. The column used was a Symmetry C18 Column, 100 Å, 3.5 μm, 4.6 mm × 75 mm (Waters), and the detector was Waters 2475 HPLC Multi Fluorescence Detector (Waters), with emission at 315 nm and excitation at 265 nm.

### Enzymatic assay

A spectrophotometric enzyme assay was used to test l-lactaldehyde dehydrogenase activity, measuring NADH formation, as described in Caballero et al. [65]. The Tecan Infinite M200 Pro plate reader was used to measure absorbance at 340 nm at 25 °C for 10 min. The test was carried out in 200 µL with 0.05 mM l-lactaldehyde, 2.5 mM NAD, 1 mM reduced glutathione, and 500 mM glycine buffer pH 10.5, crude extract from bacterial cultures. l-Lactaldehyde was used for initiating the reaction. The crude extract was obtained by mixing 1.5 mL cell suspension with 200 µL glass beads, 0.1 mm in diameter. Cells were lysed by Mixer Mill MM400 (Retsch) at 300 s^− 1^ for 1 min, for a total of 5 cycles, with samples placed on ice between cycles. One unit of enzyme activity (IU) is here defined as the amount of enzyme needed to transform 1 µmol of substrate per minute, calculated using Beer-Lambert law with extinction coefficient 6,220 M^− 1^ cm^− 1^ [66].

### Statistical analysis

All experiments were done in triplicate. Statistical analyses were conducted using IBM SPSS Statistics software (IBM) [67]. One-way analysis of variance (ANOVA) was used to assess statistical differences between groups, with Levene’s test applied to evaluate homogeneity of variances [68]. Tukey’s HSD or Games–Howell post hoc tests were used as appropriate. Differences were considered statistically significant at *p* < 0.05.

## Results

### Enabling consumption of l-rhamnose and l-fucose in *C. glutamicum*

**Fig. 2:**
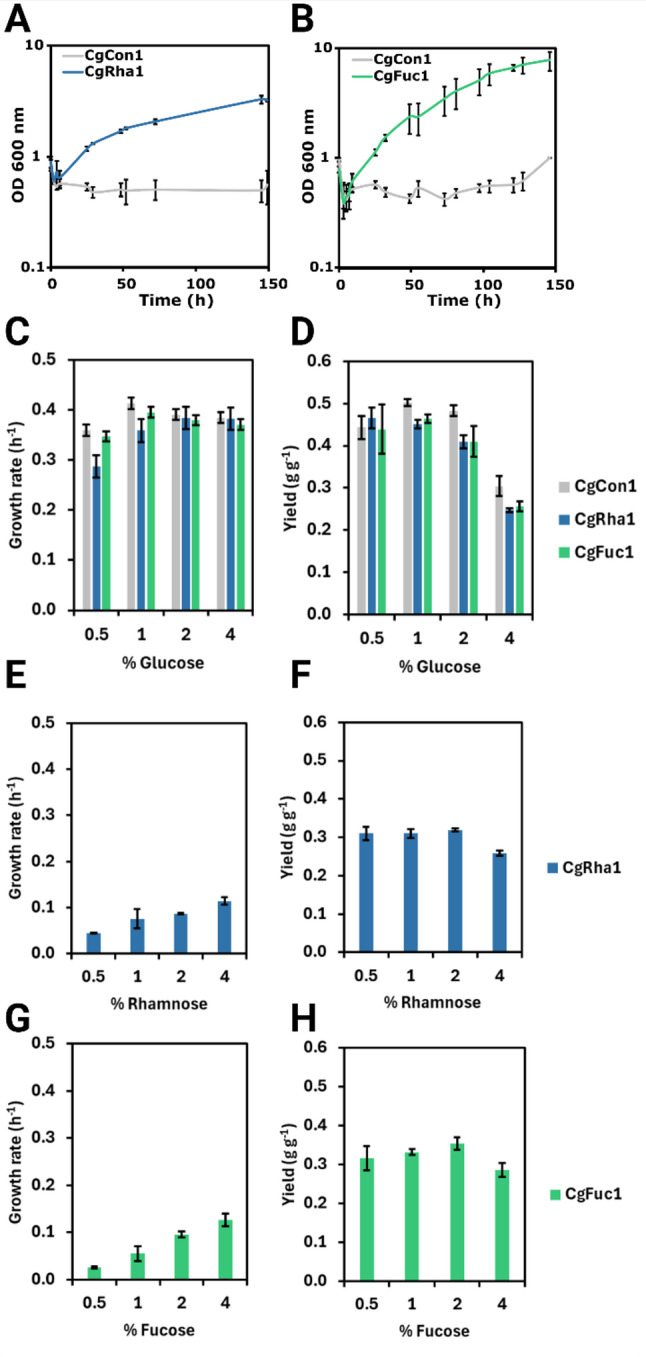
A Growth of *C. glutamicum* strains CgCon1 (grey line) and CgRha1 (blue line) in 1% l-rhamnose. B Growth of *C. glutamicum *strains CgCon1 (grey line) and CgFuc1 (green line) in 1% l-fucose. Growth rate (h^-1^) and biomass yield (g g^-1^) of CgCon1 (grey bars), CgRha1 (blue bars), and CgFuc1 (green bars) cultivated in CGXII medium supplemented with 0.5, 1, 2, or 4% d-glucose (C and D), l-rhamnose (E and F), or l-fucose (G and H) as the sole carbon source. Cultivation was performed in 250 mL shake flasks with 25 mL CGXII medium, at 30 °C and 150 rpm. Average values, standard deviations, growth rates, and biomass yields from technical triplicates are shown.

The genetic components responsible for deoxy sugar utilization in *E. coli* were harnessed to enable *C. glutamicum* to metabolize these sugars as carbon sources. Hence, the l-rhamnose-utilizing genes *rhaBADM* were overexpressed in *C. glutamicum* through the plasmid pVWEx1, yielding the strain CgRha1. Similarly, the l-fucose-utilizing genes *fucIKUA* were overexpressed in *C. glutamicum*, yielding the strain CgFuc1. The newly constructed strains, along with the control strain *C. glutamicum* (pVWEx1), named CgCon1, were cultivated in CGXII minimal medium supplemented with 1% l-rhamnose or l-fucose as the sole carbon source. While the empty-plasmid control strain CgCon1 did not grow on l-rhamnose or l-fucose, the strain CgRha1 grew on l-rhamnose (Fig. 2A), and the strain CgFuc1 was able to grow on l-fucose as the sole carbon source (Fig. 2B). Additionally, to assess substrate specificity, CgRha1 was cultivated in CGXII medium with 1% l-fucose, and CgFuc1 in CGXII medium with 1% l-rhamnose. However, no growth was observed under the conditions tested (data not shown).

Furthermore, the impact of gradually increasing carbon source concentrations on the growth of the engineered *C. glutamicum* strains was evaluated. The CgRha1 and CgFuc1 strains were cultivated in CGXII medium supplemented with l-rhamnose and l-fucose, respectively, at concentrations of 0.5%, 1%, 2%, or 4%. In addition, d-glucose was used at the same concentrations as a control carbon source. For d-glucose, the growth rate values were similar across the tested strains for concentrations between 1 and 4% (Fig. 2C). The biomass yields obtained during cultivation in 4% d-glucose showed a decrease for all strains, CgCon1, CgRha1, and CgFuc1, with a reduction of 0.18 ± 0.02, 0.16 ± 0.02, and 0.15 ± 0.04 g g^− 1^, respectively, in comparison to 2% d-glucose (Fig. 2D). In contrast, the biomass yield remained constant across growth with different concentrations of l-rhamnose and l-fucose, with a small decrease of 0.06 ± 0.02 g g^− 1^ from 2 to 4% for both l-rhamnose and l-fucose (Fig. 2F and H). However, with both l-rhamnose and l-fucose, increased sugar concentrations correlated with higher growth rates. Specifically, CgRha1 cultivated in CGXII medium with 4% l-rhamnose achieved a growth rate of 0.11 ± 0.01 h^− 1^ (Fig. 2E) while CgFuc1 cultivated with 4% l-fucose exhibited a similar growth rate of 0.13 ± 0.01 h^− 1^ (Fig. 2G). Consumption of 96–100% of the tested sugars by the strains CgCon1, CgRha1, and CgFuc1 was confirmed by HPLC analysis of culture supernatants.

### Overexpression of permeases for improved growth of *C. glutamicum* on deoxy sugars

**Fig. 3:**
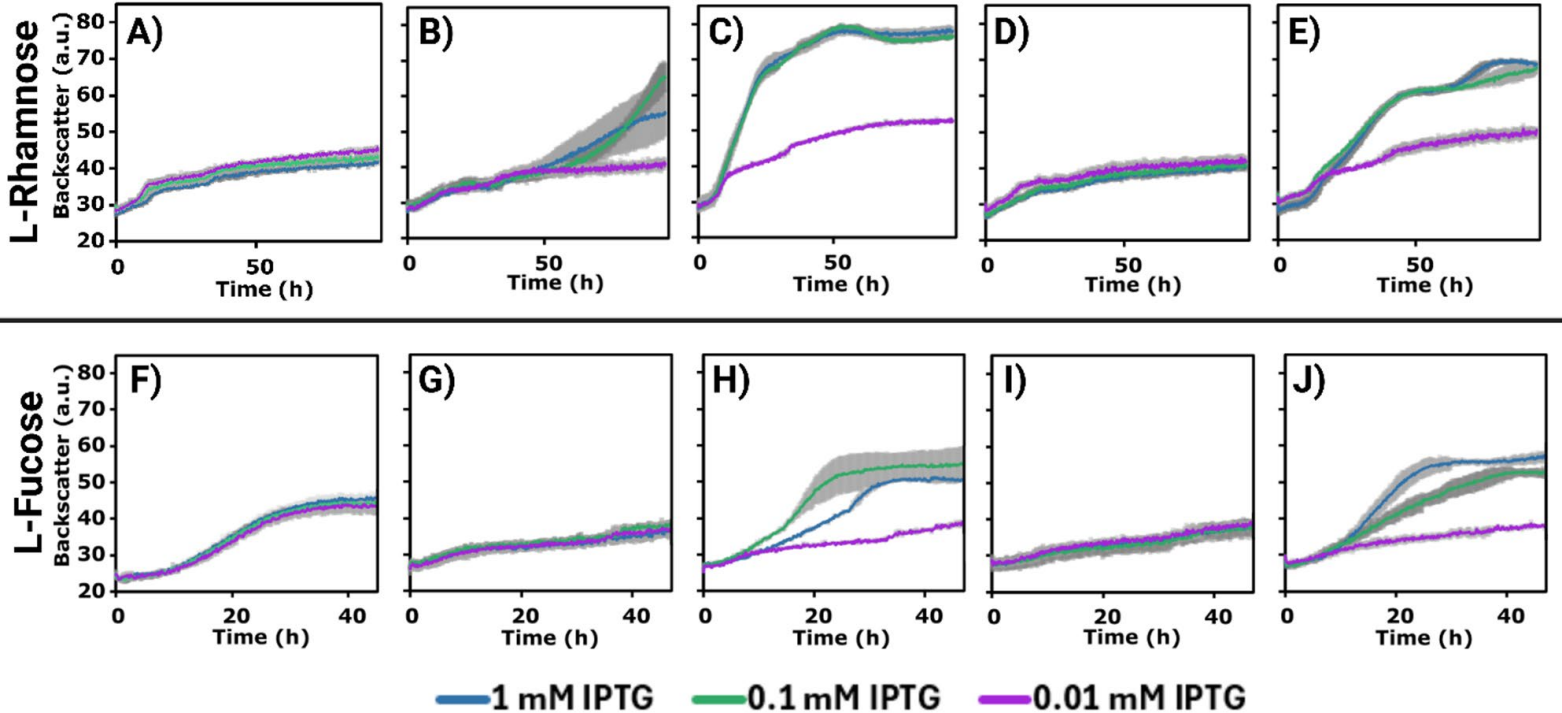
Growth curves from microbioreactor cultivations with CGXII medium for the strains CgRha1C (**A**), CgRha2 (**B**), CgRha3 (**C**), CgRha4 (**D**), and CgRha5 (**E**) supplemented with 1% l-rhamnose and of the strains CgFuc1C (**F**), CgFuc2 (**G**), CgFuc3 (**H**), CgFuc4 (**I**), and CgFuc5 (**J**) grown with 1% l-fucose. The expression of the cloned genes was induced by adding 1 mM (blue lines), 0.1 mM (green lines), or 0.01 mM (purple lines) IPTG. Average values and standard deviations from technical triplicates are shown

To assess the uptake of the non-native deoxy sugars l-rhamnose and l-fucose and to improve microbial growth rates, four different permeases were evaluated. The transport proteins investigated were RhaT and FucP, both from *E. coli* gDNA, and IolT1 and IolT2, both from *C. glutamicum* gDNA. Hence, the plasmids pECXT99a, pECXT99a-*rhaT*, pECXT99a-*fucP*, pECXT99a-*iolT1*, and pECXT99a-*iolT2* were transferred to the strain CgRha1, yielding the strains CgRha1C, CgRha2, CgRha3, CgRha4, and CgRha5; and to the strain CgFuc1, resulting in the strains CgFuc1C, CgFuc2, CgFuc3, CgFuc4, and CgFuc5 (Table 2). The newly constructed strains were cultivated in 1 mL CGXII cultures in a microbioreactor at 30 °C at 1,100 rpm (Fig. 3). Varying induction levels were supplemented in the medium with IPTG at the final concentrations of 1 mM, 0.1 mM, or 0.01 mM (Fig. 3). The strains CgRha1C and CgFuc1C were used as controls (Fig. 3A and F).

For growth on l-rhamnose, the strain CgRha2 overexpressing *rhaT* initiated growth only after a lag phase of approximately 70 h under both 1 and 0.1 mM IPTG (Fig. 3B). In contrast, the strain CgRha4 overexpressing *iolT1* did not grow with any IPTG induction levels tested (Fig. 3D). For the CgRha3 and CgRha5, overexpressing *fucP* and *iolT2*, respectively, induction with 0.01 mM IPTG resulted in a lower final biomass compared to the strains induced with higher IPTG concentrations (Fig. 3C and E). With *fucP* overexpression, the final biomass was reached after approximately 50 h when induced with either 1 mM or 0.1 mM IPTG (Fig. 3C). In comparison, *iolT2* overexpression resulted in the final biomass being reached after approximately 70 h when induced with 1 mM IPTG (Fig. 3E). Notably, both 0.1 mM and 1 mM IPTG resulted in the same final biomass (Fig. 3C and E).

As for growth on l-fucose, strains overexpressing *rhaT* and *iolT1* (Fig. 3G and I) showed slow growth regardless of the level of IPTG and ended up with lower final biomass compared with CgFuc1C. For the CgFuc3 strain overexpressing *fucP*, the final biomass was achieved after 30 h with 1 mM induction (Fig. 3I). In contrast, for the CgFuc5 strain with *iolT2* overexpression, the final biomass was reached after 24 h of growth, also with 1 mM induction (Fig. 3J).

These cultivations were extended to 95 and 45 h for growth on l-rhamnose and l-fucose, respectively, to check the final biomass for the various induction levels (Fig. 3). This extended cultivation allowed us to conclude that 1 mM IPTG is preferable over 0.1 mM IPTG for CgFuc5 (Fig. 3J). Based on the final biomass and the time required to reach the plateau, we proceeded with CgRha3, overexpressing *fucP* for l-rhamnose transport, and CgFuc5, overexpressing *iolT2* for l-fucose transport, as they showed a 3.1-fold and 2.8-fold faster growth, respectively, compared with the control strains CgRha1C and CgFuc1C (Fig. 3A and F).

Both the utilization operons and the preferred transporter genes were combined into the pVWEx1 and pSH1 plasmids, with the latter carrying a constitutive P_*tuf*_ promoter system for continuous gene expression (Table 1). Therefore, the plasmids pVWEx1-*rhaBADM-fucP*, pVWEx1-*fucIKUA-*iolT2, pSH1-*rhaBADM-fucP*, and pSH1-*fucIKUA-iolT2* were constructed and transferred to wild-type *C. glutamicum*, yielding the strains CgRha6, CgFuc6, CgRha7, and CgFuc7 (Table 2).

**Fig. 4:**
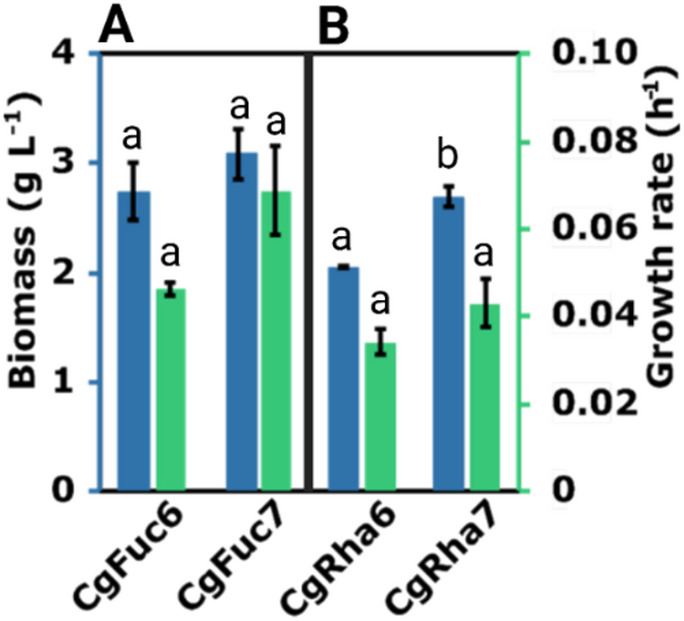
Growth rates and final biomass of *C. glutamicum* strains CgFuc6 and CgFuc7 in 1% l-fucose (**A**). CgRha6 and CgRha7 in 1% l*-*rhamnose (**B**). Average values and standard deviations from technical triplicates are shown. Different letters represent significant differences as decided with a one-way ANOVA test. Between CgFuc6 and CgFuc7, there are no significant changes in either biomass or growth rate. Difference between CgRha6 and CgRha7: Biomass = significant (*p* = 0.019), growth rate = non-significant

These newly constructed strains were cultivated in CGXII medium in 250 mL baffled flasks supplemented with 1% carbon source, either l-rhamnose or l-fucose. According to our statistical analysis, neither the final biomass nor the growth rate was significantly different when comparing CgFuc6 and CgFuc7 (Fig. 4). However, when comparing CgRha6 and CgRha7, a higher final biomass for CgRha7 (2.69 g L^− 1^ ± 0.10 g L^− 1^) was observed in comparison to CgRha6 (2.05 g L^− 1^ ± 0.01 g L^− 1^), corresponding to approximately 31% increase when using the pSH1 vector (*p* = 0.019) (Fig. 4). Hence, the pSH1 vector was selected for further experiments.

### Identification of a native lactaldehyde dehydrogenase in *C. glutamicum*

**Fig. 5:**
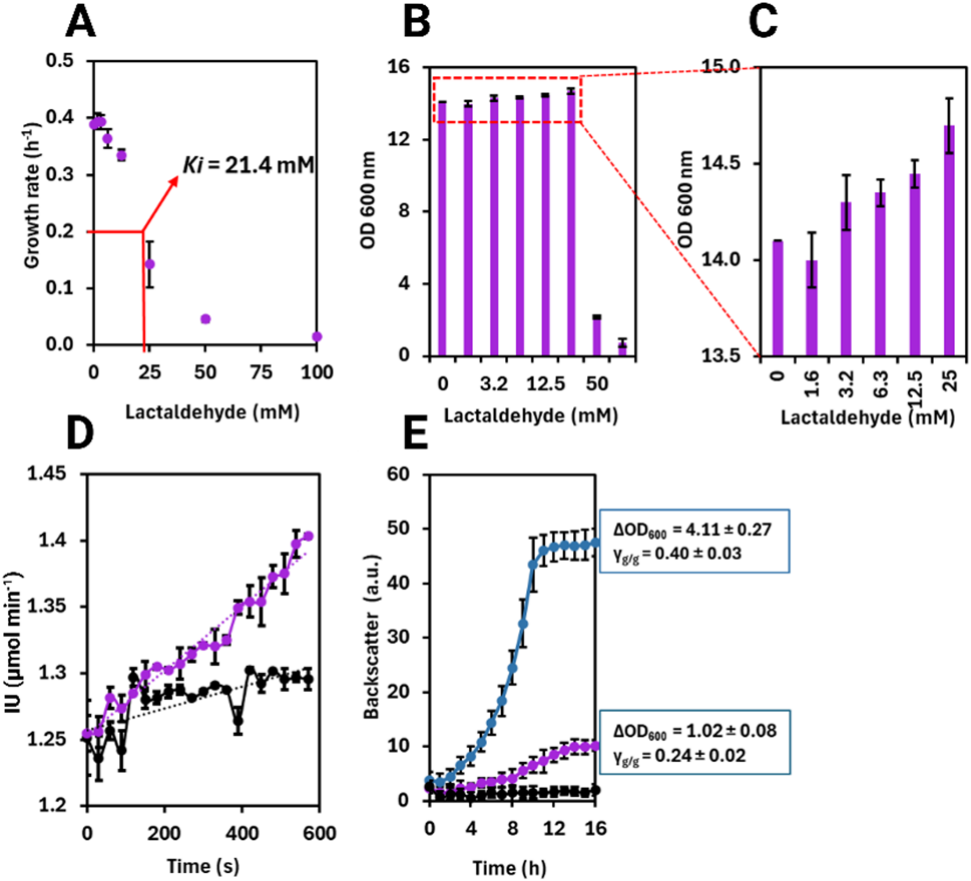
Growth rates of *C. glutamicum* wild-type in 1% glucose minimal medium supplemented with 0, 1.56, 3.13,6.25, 25, 50, or 100 mM of l-lactaldehyde (**A**). Final OD600 values of *C. glutamicum* wild-type in 1% glucose minimal medium supplemented with 0, 1.56, 3.13, 6.25, 25, 50, or 100 mM of l-lactaldehyde (**B** and **C**). Lactaldehyde dehydrogenase activity, using an NADH assay and crude extract of *C. glutamicum* wild-type cultivated with D-glucose as the sole carbon source. Purple data: l-lactaldehyde included in the reaction. Black data: l-lactaldehyde not included in the reaction (**D**). Growth curve of *C. glutamicum* wild-type cultivated with 20 mM D-glucose (blue), 20 mM l-lactaldehyde (purple), and no carbon source (black) (**E**). Average values and standard deviations from technical triplicates are shown

As an intermediate of the l-rhamnose and l-fucose utilization pathways, l-lactaldehyde is formed (Fig. 1). To study the effect of l-lactaldehyde in *C. glutamicum*, the wild-type strain was cultivated in 1% glucose minimal medium supplemented with different concentrations of l-lactaldehyde ranging from 0 to 100 mM. The inhibition constant (K_i_) was determined to be 21.4 mM (Fig. 5A). Additionally, biomass values were collected (Fig. 5B), and slight increase changes in final biomass were observed in correspondence with increasing l-lactaldehyde supplementation, with the most pronounced changes occurring at the highest l-lactaldehyde concentrations (Fig. 5C). These observations indicated a previously unrecognized metabolic activity in *C. glutamicum*. Therefore, the native lactaldehyde dehydrogenase activity of wild-type *C. glutamicum* crude extracts was examined using the NADH-formation assay. The assay showed increased absorbance at 340 nm upon addition of l-lactaldehyde to the assay mixture (Fig. 5D). Next, the growth of *C. glutamicum* wild-type was evaluated in minimal medium supplemented with 20 mM d-glucose, 20 mM l-lactaldehyde, or no carbon source. The results showed a biomass yield on d-glucose of 0.40 ± 0.03 g g^− 1^ and on l-lactaldehyde of 0.24 ± 0.02 g g^− 1^ (Fig. 5E).

**Fig. 6:**
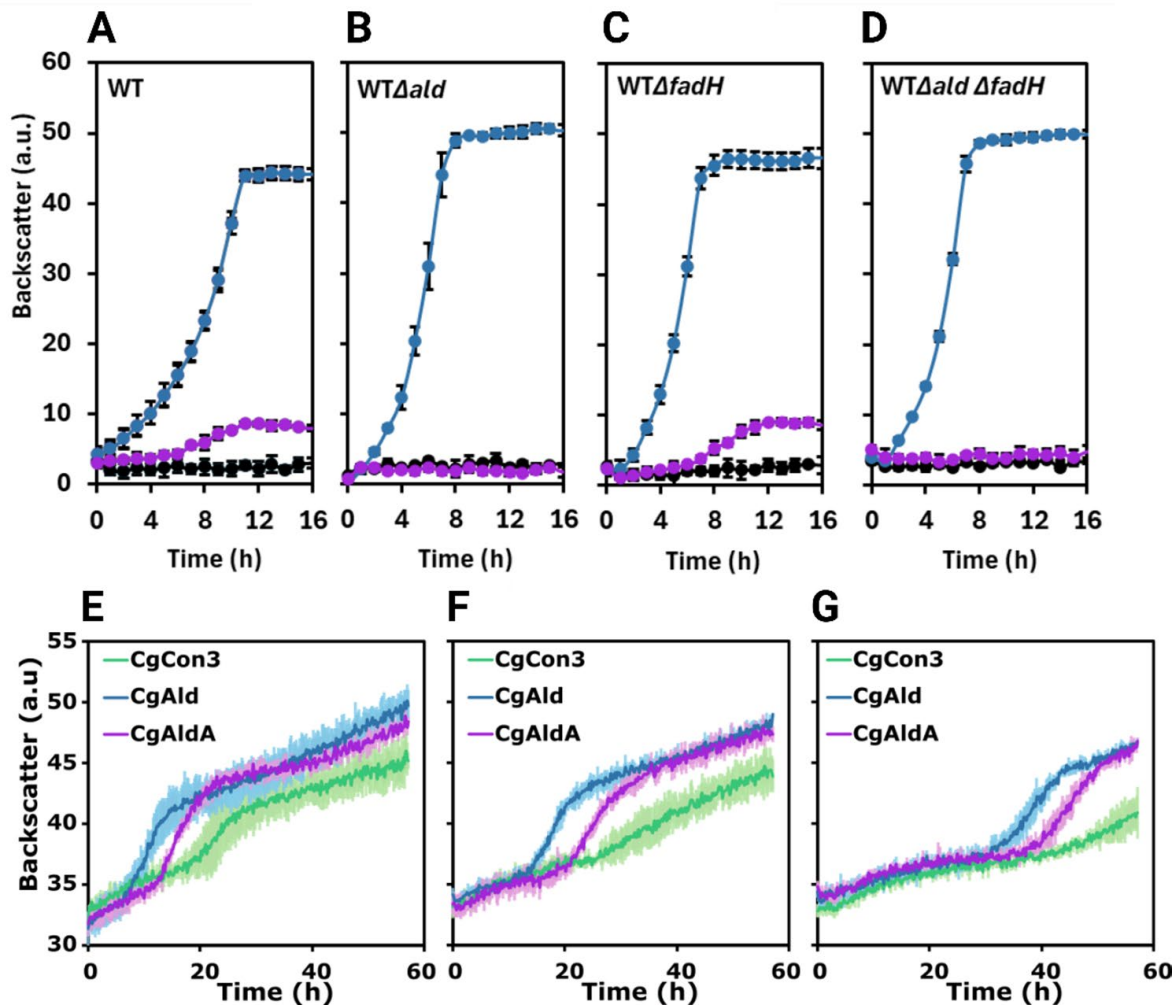
Growth curves of *C. glutamicum* wild-type (WT) (**A**), WTΔ*ald* (**B**), WTΔ*fadH* (**C**), and WT*ΔaldΔfadH* (**D**). The blue curves represent growth on 20 mM d-glucose, the purple curves represent growth on 20 mM l-lactaldehyde, while the black curves represent growth with no carbon source. Comparison of the growth of CgCon3, CgAld, and CgAldA with 20 mM (**E**), 30 mM (**F**), and 40 mM (**G**) l-lactaldehyde as the sole carbon source. Average values and standard deviations from technical triplicates are shown

**Fig. 7:**
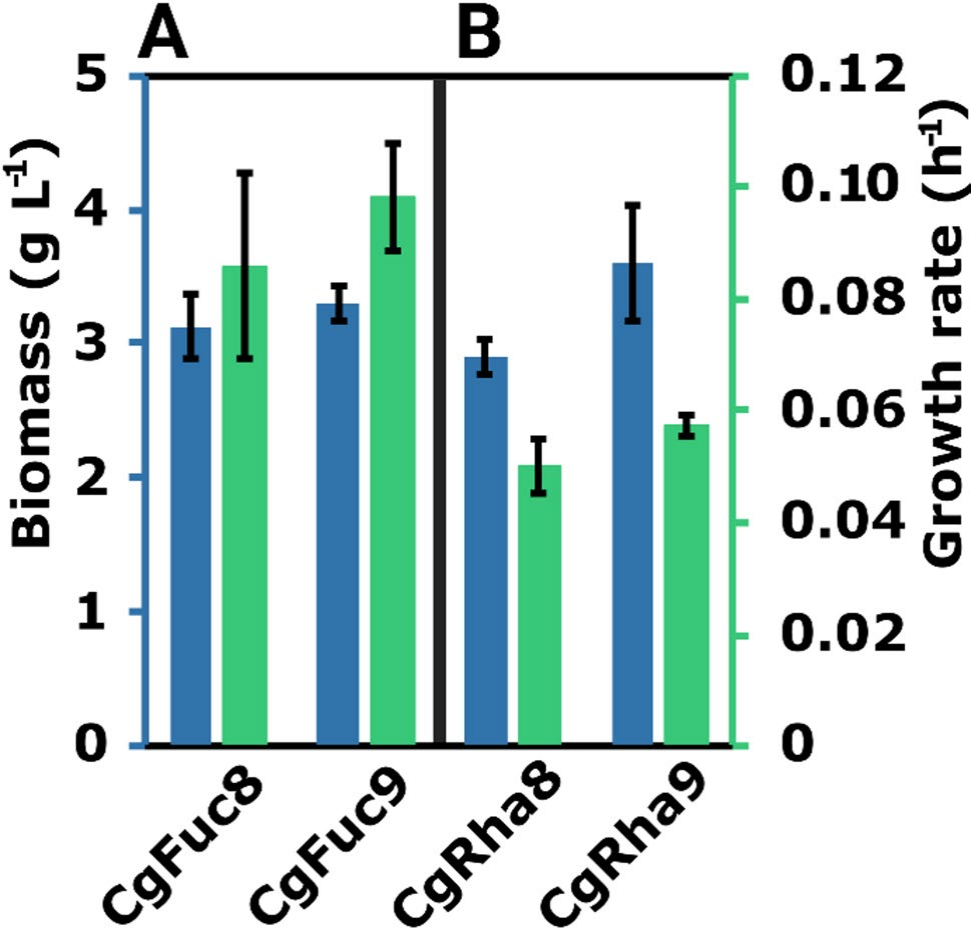
Growth rates and final biomass of *C. glutamicum* strains (**A**) CgFuc8 and CgFuc9 in 1% l-fucose. (**B**) CgRha8 and CgRha9 in 1% l*-*rhamnose. Average values and standard deviations from technical triplicates are shown. No significant differences were found using the one-way ANOVA test (*p* = 0.052)

It is known that *C. glutamicum* possesses acetaldehyde and formaldehyde dehydrogenases encoded by *ald* and *fadH*, respectively [61]. To determine whether either enzyme also exhibits lactaldehyde dehydrogenase activity, the strains *C. glutamicum* wild-type, WT*Δald*, WT*ΔfadH*, and WT*ΔaldΔfadH* were cultivated in minimal medium containing either 20 mM d-glucose, 20 mM l-lactaldehyde, or no carbon source. Under these conditions, WT*Δald* and WT*ΔaldΔfadH* did not grow in the presence of l-lactaldehyde, indicating that the acetaldehyde dehydrogenase encoded by *ald* also catalyzes l-lactaldehyde oxidation (Fig. 6A-D). To assess whether overexpression of a lactaldehyde dehydrogenase could result in faster growth of *C. glutamicum* during utilization of l-lactaldehyde, the strains CgAldA, overexpressing *aldA* from *E. coli*, and CgAld, overexpressing *ald* from *C. glutamicum*, were cultivated in minimal medium containing either 20, 30, or 40 mM of l-lactaldehyde as the sole carbon source. The strain CgCon3 was used as a control. The results showed that overexpression of *aldA* or *ald* in *C. glutamicum* enabled faster growth at the tested l-lactaldehyde concentrations compared to the strain not overexpressing any dehydrogenase (CgCon3). However, the native *ald* outperformed *aldA* **(**Fig. 6E-G**)**. Finally, the best-performing strains growing on l-rhamnose (CgRha7) and l-fucose (CgFuc7) were transformed with pECXT99a and pECXT99a-*ald*, yielding CgRha8 and CgRha9, and CgFuc8 and CgFuc9. These strains were grown in 1% l-rhamnose or l-fucose (Fig. 7). The observed changes in growth rate and biomass in strains harboring *ald* did not reach statistical significance (*p* = 0.052). Therefore, *ald* was included in subsequent growth experiments using seaweed hydrolysates as the cultivation medium in order to prioritize homologous expression.

Pathway engineering enables increa

sed conversion of l-lactaldehyde into central carbon metabolites, enhancing deoxy sugar-based growth. Ald was identified as the optimal lactaldehyde dehydrogenase in *C. glutamicum.*

### Engineering *C. glutamicum* for growth on brown and green seaweed hydrolysate and l-lysine production

**Fig. 8:**
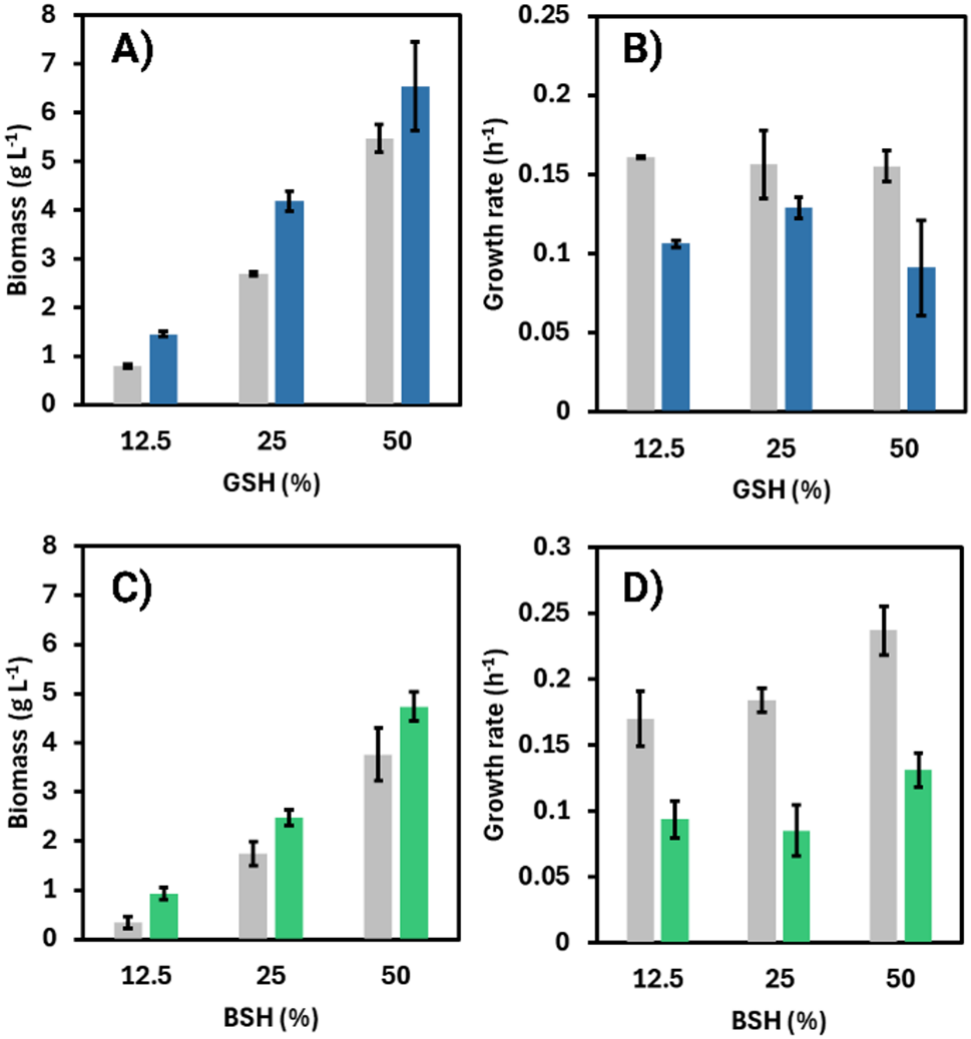
Green seaweed-based growth of strains CgCon4 (grey bars) and CgRha9 (blue bars) with different concentrations of seaweed hydrolysate from *U. lactuca*, growth is represented as biomass (**A**) and growth rate (**B**). Brown seaweed-based growth of strains CgCon4 (grey bars) and CgFuc9 (green bars) with different concentrations of seaweed hydrolysate from *S. latissima*, growth is represented as biomass (**C**) and growth rate (**D**). Average values and standard deviations from technical triplicates are shown

Biomass from *U. lactuca* and *S. latissima* was used to prepare GSH and BSH, respectively, as described in Methods 2.3. The compositions of the resulting hydrolysate mixtures were analyzed by HPLC. In GSH, d-glucose (16.04 g L^− 1^), l-rhamnose (9.14 g L^− 1^), d-xylose (0.25 g L^− 1^), and d-fructose (0.72 g L^− 1^) were detected, whereas in BSH, d-glucose (9.98 g L^− ^1), l-fucose (3.57 g L^− 1^), d-mannitol (15.48 g L^− 1^), and d-fructose (0.69 g L^− 1^) were identified. Other compounds that can promote growth in *C. glutamicum* as carbon sources such as acetate, ethanol or lactic acid were not detected. Formic acid, methanol, furfural and 5HMF are known growth inhibitors commonly present in this type of hydrolysate; however, no detectable amounts of these compounds were found in the hydrolysates. Next, the strains CgRha9 and CgFuc9 were tested using seaweed hydrolysate from *U. lactuca* and *S. latissima*, respectively, as the carbon source. The strain CgCon4 served as a control carrying the empty plasmids pSH1 and pECXT99a (Table 2). These strains were cultivated in a microbioreactor in various concentrations of seaweed hydrolysate diluted in CGXII medium (12.5%, 25%, and 50%). Under all the conditions tested, CgCon4 showed higher growth rates compared to the engineered strains. However, CgRha9 and CgFuc9 presented higher final biomass values compared to CgCon4. CgRha9 showed biomass increases of 84%, 55%, and 20% compared to CgCon4 for 12.5%, 25%, and 50% GSH, respectively. CgFuc9 showed biomass increases of 175%, 43%, and 26% compared to CgCon4 for 12.5%, 25%, and 50% BSH, respectively. HPLC analysis of supernatants confirmed 82–89% and 90–96% consumption of l-rhamnose and l-fucose, respectively, by the engineered strains, consistent with their higher final biomass relative to the control (Table 3). This consumption of l-rhamnose corresponds to 15–20% of the total biomass for CgRha9, and l-fucose consumption accounts for 11–14% of the total biomass formation for CgFuc9 when cultivated in seaweed hydrolysate (Table 3). Furthermore, no l-lactaldehyde was detected in either sample supernatant, therefore it is assumed to have been consumed by the cells (Fig. 8).

**Fig. 9:**
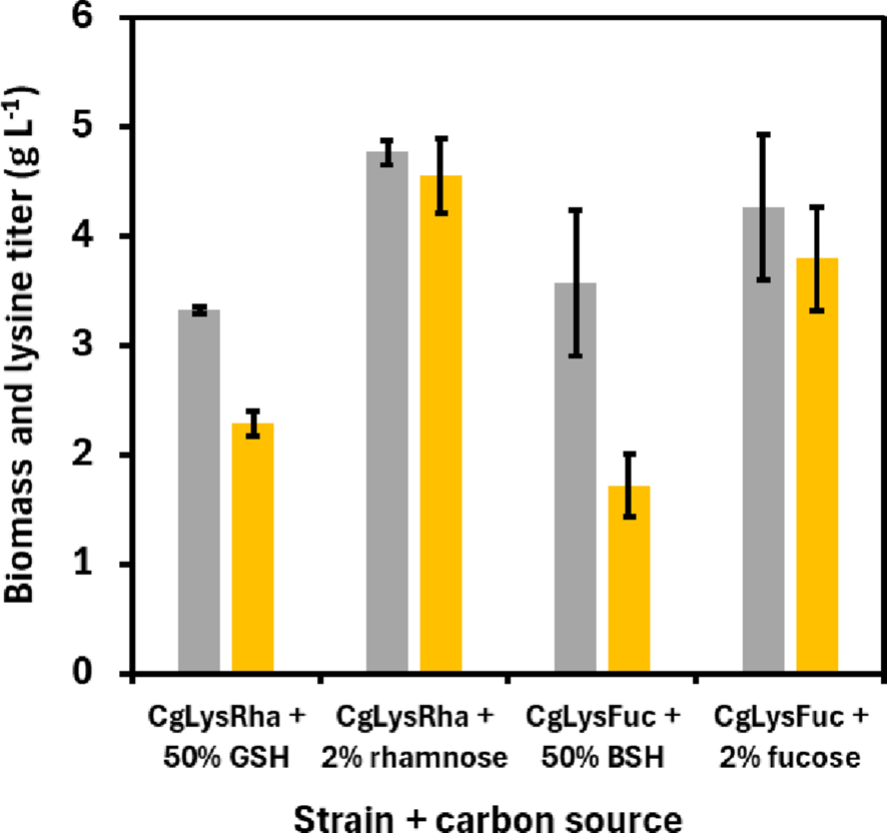
Biomass formation (grey bars) and l-lysine production (yellow bars), expressed in g L^− 1^, by strain CgLysRha using 50% GSH or 2% l-rhamnose as the carbon source, and by strain CgLysFuc using 50% BSH or 2% l-fucose as the carbon source. Average values and standard deviations from technical triplicates are shown

To demonstrate the industrial relevance of these findings, the l-lysine producing strain GSLA [60] was transformed with the plasmids pSH1-*rhaBADM-fucP* and pECXT99a-*ald*, as well as pSH1-*fucIKUA-iolT2* and pECXT99a-*ald* to obtain the strains CgLysRha and CgLysFuc, respectively. The strains CgLysRha, and CgLysFuc were cultivated in a BioLector microbioreactor at 30 °C and 1,100 rpm. Supernatants were harvested and analyzed with HPLC for l-lysine content. The strains reached l-lysine titers of 4.6 g L^− 1^, 3.8 g L^− 1^, 2.3 g L^− 1^ and 1.7 g L^− 1^ from 20 g L^− 1^ l-rhamnose, 20 g L^− 1^ l-fucose, 50% GSH and 50% BSH as the carbon source, respectively (Fig. 9).

**Table 3:** Utilization of the deoxy sugars l-fucose and l-rhamnose by the strains CgFuc9 and CgRha9.

SWH	Strain	Used deoxy sugar (%)	Biomass from deoxy sugar (g L^− 1^)	Biomass from deoxy sugar (%)	Total yield from SWH (g g^− 1^)
BSH 50%	CgFuc9	94.4 ± 0.6	0.54 ± 0.05	11.4 ± 0.1	0.66 ± 0.04
BSH 25%	CgFuc9	97.1 ± 0.2	0.28 ± 0.03	11.2 ± 0.1	0.70 ± 0.05
BSH 12.5%	CgFuc9	94.9 ± 0.2	0.14 ± 0.01	14.6 ± 0.2	0.52 ± 0.06
GSH 50%	CgRha9	87.8 ± 12.5	1.24 ± 0.13	19.0 ± 0.6	0.51 ± 0.07
GSH 25%	CgRha9	89.4 ± 0.6	0.63 ± 0.06	15.1 ± 0.1	0.65 ± 0.03
GSH 12.5%	CgRha9	82.2 ± 0.3	0.29 ± 0.03	20.0 ± 0.1	0.45 ± 0.02

Total yield is calculated based on the available carbohydrates for the specific strain

## Discussion

Sustainable alternative feedstocks for microbial growth and production of value-added compounds are a developing field due to the increased pressure on the world’s resources [2]. By expanding the substrate spectrum of the industrially important microorganism *C. glutamicum*, we work strategically towards enabling seaweed biorefineries, where most of the available biomass is used.

In this work, two heterologous pathways, encoded by the operons *rhaBADM* and *fucIKUA*, have successfully been introduced to *C. glutamicum* for the first time utilization of l-rhamnose and l-fucose, respectively, with *E. coli* as the gene donor. Although *E. coli* can use these sugars natively [69], *C. glutamicum* offers distinct industrial advantages. Unlike *E. coli*, *C. glutamicum* lacks a strict catabolite repression system, allowing it to consume some carbon sources simultaneously [52]. It has been studied to produce various products, such as amino acids and vitamins [29, 51, 57, 70, 71]. Furthermore, *C. glutamicum* is used in a million-ton industry to produce l-glutamate and l-lysine, underscoring its robustness as a large-scale biocatalyst and highlighting its potential for sustainable bioproduction from renewable carbon sources [45, 72].

Growth was achieved on l-rhamnose and l-fucose with the engineered strains, although the observed growth rates were significantly lower than those on d-glucose (Fig. 2). For CgRha1, the growth rate decreased to 0.11 ± 0.01 h^− 1^ when using l-rhamnose as the sole carbon source, compared to 0.38 ± 0.02 h^− 1^ on d-glucose. For CgFuc1, the growth rate was measured to be 0.13 ± 0.01 h^− 1^ cultivated on l-fucose compared to 0.37 ± 0.01 h^− 1^ on d-glucose (Fig. 2). Metabolomic and transcriptomic analyses of *E. coli*, investigating the differential response between l-fucose and d-glucose metabolism, have been performed [73]. It was found that ATP depletion was associated with l-fucose metabolism, primarily due to upregulated gluconeogenesis, compared to d-glucose metabolism [73]. Furthermore, ATP deficiency can hinder FucK and RhaB from phosphorylating the substrate and lead to reduced flux of catabolites and slower growth [74–76]. To address this metabolic challenge and, more broadly, to expand microbial substrate spectra, strategies such as improving substrate uptake, pathway regulation, cofactor and redox balancing, and overall growth performance can enhance the conversion of new substrates into biomass or products [77–79]. In this context, adaptive laboratory evolution (ALE) represents a particularly useful approach [80].

Nevertheless, the growth observed upon overexpression of only the catabolic operons *rhaBADM* and *fucIKUA* in *C. glutamicum* indicated that this bacterium possesses native transporter systems for these sugars (Fig. 2). *C. glutamicum* uses primarily three phosphoenolpyruvate-dependent phosphotransferase systems (PTS) to import and phosphorylate the sugars d-glucose, d-fructose, and d-sucrose using phosphoenolpyruvate as phosphoryl donor [49, 81, 82]. Furthermore, the native myo-inositol/proton symporters IolT1 and IolT2 work as secondary transport system for d-glucose which is then phosphorylated by the glucose kinase GlK using ATP or by the polyphosphate/ATP-dependent glucokinase PpgK using ATP or Poly phosphates [83–85]. In addition, IolT1 facilitates the uptake of d-xylose, whereas IolT2 facilitates the uptake of d-galactose [30, 86]. Together, these findings indicate substrate flexibility among these permeases. To investigate the transport of l-rhamnose and l-fucose, the two native transporters, IolT1 and IolT2, and the two non-native transporters from *E. coli*, RhaT and FucP, were evaluated in this study. The results showed that IolT2 functions as a permease for both l-rhamnose and l-fucose, whereas IolT1 does not (Fig. 3). Unexpectedly, FucP outperformed RhaT for L-rhamnose uptake under the conditions tested. This may reflect the broader substrate specificity of FucP, which could facilitate transport of structurally related deoxy sugars. Moreover, in a study, FucP was shown to participate in import of d-fructose in *E. coli*, supporting the previous claim [87]. Membrane transport proteins often exhibit unpredictable behaviors when cloned and expressed in non-native hosts, and substrate specificity in sugar transporters can vary substantially depending on protein context and host physiology [88].

In *E. coli*, l-rhamnose and l-fucose can serve as carbon and energy sources via phosphorylating pathways [42, 89]. During their catabolism, glycerone phosphate and l-lactaldehyde are formed. The first enters glycolysis whereas the further conversion of l-lactaldehyde is influenced by oxygen availability. Under anaerobic conditions, l-lactaldehyde is mainly converted by FucO to 1,2-propanediol, while in the presence of oxygen, it is oxidized by AldA to lactate and can subsequently enter central carbon metabolism [90]. An alternative non-phosphorylating pathway has been described for l-rhamnose in *Pichia stipitis* where catabolism of this sugar yields l-lactaldehyde and pyruvate instead [91]. In this research, the growth of wild-type *C. glutamicum* on l-lactaldehyde as the sole carbon source was observed (Fig. 5E) and Ald was identified as a lactaldehyde dehydrogenase in *C. glutamicum* (Fig. 6B). Ald, encoded by *ald*, has previously been identified as an acetaldehyde dehydrogenase involved in ethanol catabolism via oxidation of acetaldehyde [92]. In addition, Ald also participates in the detoxification of formaldehyde together with FadH [61]. The ability of the acetaldehyde dehydrogenase Ald to oxidize l-lactaldehyde can be rationalized by the chemical similarity between acetaldehyde and l-lactaldehyde. Although l-lactaldehyde is a C3 compound, both molecules are short-chain aliphatic aldehydes with a carbon backbone beyond the aldehyde group, in contrast to formaldehyde, which lacks such a backbone. Aldehyde dehydrogenases are known to exhibit substrate promiscuity toward structurally related short-chain aldehydes, which likely enables Ald to accommodate l-lactaldehyde as an alternative substrate in *C. glutamicum* [93].

In the last part of this study, the engineered *C. glutamicum* strains were cultivated using GSH and BSH as carbon sources. The seaweed hydrolysates were obtained via sulfuric acid hydrolysis and cellulase-mediated depolymerization, which have proven to be effective methods to break down the polymers to more available monomers [94–96]. In a study by Adasteinsson et al. the researchers identified several fucoidanases and ulvan lyases that could enhance the degradation of the sulphated fucoidan and ulvan present in brown and green seaweeds, respectively [97]. However, based on the detected carbohydrates in this study, l-rhamnose accounts for 35% of the total, while fucose accounts for 12%, consistent with previously reported compositional values [37–41]. Engineered *C. glutamicum* has previously been cultivated on hydrolysates derived from the brown seaweeds *Laminaria hyperborea*,* Laminaria digitata*, and *Durvillaea antarctica* [29, 70]. In addition, the growth of engineered *C. glutamicum* on red seaweed hydrolysate (RSH) from *Palmaria palmata* has also been reported [30]. To our knowledge, this study represents the first use of green seaweed as a carbon source for *C. glutamicum*. This finding highlights the potential of green seaweed as a feedstock, given its high carbohydrate content and widespread availability [98, 99]. When using 40% BSH from *L. hyperborea* as a carbon source with the engineered *C. glutamicum* strain utilizing d-glucose and d-mannitol, the achieved biomass yield was approximately 0.70 g g^− 1^ [29]. Similarly, when using 50% RSH from *P. palmata* as a carbon source with an engineered *C. glutamicum* strain utilizing d-glucose, d-galactose, and d-xylose, the achieved biomass yield was approximately 0.40 g g^− 1^ [30]. In comparison, the engineered strains of this work reached biomass yields of 0.66 g g^− 1^ and 0.50 g g^− 1^ when cultivated on 50% BSH and GSH, respectively (Table 3).

Growth inhibitors commonly found in hydrolysates include furfural and 5HMF [30, 32]. However, these compounds were not detected in the hydrolysates tested in this study using the applied analytical method. Moreover, *C. glutamicum* has been reported to show high tolerance toward these types of inhibitors, which is partially attributed to its ability to detoxify furfural [100, 101]. Elemental composition of *S. latissima*, including heavy metals, has been reported by Arlov et al. [17]. They found maximum levels of 0.81 ± 0.01 mg kg^− 1^ DW cadmium (Cd), 71.3 ± 3.4 mg kg^− 1^ DW arsenic (As), and 0.30 ± 0.12 mg kg^− 1^ DW lead (Pb) [17]. Reported bacterial tolerances vary widely. *Enterobacter* sp. tolerates 540–1,220 mg L^− 1^ Cd, while *Stenotrophomonas maltophilia* tolerates around 120 mg L^− 1^, and several marine bacteria tolerate around 100 mg L^− 1^ Pb [102–104]. Other species, including *Alcaligenes faecalis*, *Bacillus* sp., *Pseudomonas aeruginosa*, and *Brevibacterium iodinium*, can detoxify mercury (Hg), Cd, and Pb, and engineered *E. coli* can bioaccumulate Hg [104, 105]. However, no tolerance data exists for As, nor are there specific heavy-metal toxicity studies for *C. glutamicum*. Heavy metals can constitute growth-inhibitory effects and, especially As, warrant further investigation to confirm *C. glutamicum* tolerance.

Additionally, to growth of deoxy sugars and seaweed hydrolysates, production of l-lysine was included in this work as a demonstrator. *C. glutamicum* is the dominant industrial workhorse for this amino acid and, worldwide l-lysine production exceeds 2.5 million tons annually, making it one of the largest biotechnological processes [106]. Driven primarily by the animal feed industry, l-lysine from alternative and eco-friendly feedstocks is of great interest. In this work, the l-Lysine producing *C. glutamicum* GSLA strain was used as a host to enable utilization of l-rhamnose, l-fucose, and l-lactaldehyde. Previously, l-lysine production of 4.8 g L^− 1^ has been reported for GSLA using 20 g L^− 1^ glucose as the carbon source [60]. In this work, 4.6 g L^− 1^ and 3.8 g L^− 1^ of l-lysine were produced from l-rhamnose and l-fucose as carbon source, respectively. Additionally, 2.3 g L^− 1^ and 1.7 g L^− 1^ of l-lysine were produced from 50% GSH and 50% BSH as carbon source, respectively. To compare, the strain *C. glutamicum* strain CgRedLys reached 4.2 g L^− 1^ of l-lysine using 75% red seaweed hydrolysate from *P. palmata* [30].

## Conclusions

In conclusion, this study demonstrates, for the first time, that *C. glutamicum* can utilize l-rhamnose and l-fucose as carbon sources via the heterologous expression of *E. coli*-derived genes. Among the tested sugar transporters, FucP supported the most efficient l-rhamnose uptake, while IolT2 was optimal for l-fucose, resulting in the highest biomass and growth rate values. We further identified Ald as a native lactaldehyde dehydrogenase in *C. glutamicum*, enabling growth on l-lactaldehyde. Seaweed hydrolysate from *U. lactuca* and *S. latissima* proved to be promising carbon sources, providing substantial amounts of both l-rhamnose and l-fucose in addition to other fermentable carbohydrates. Finally, l-lysine production with the engineered strains using l-rhamnose, l-fucose, BSH, or GSH as carbon source was demonstrated. Collectively, these findings provide a foundation for future studies aiming to exploit *C. glutamicum* in brown and green seaweed-based bioprocesses.

## Supplementary Information

Below is the link to the electronic supplementary material.

Supplementary Material 1.

## Data Availability

All data supporting the findings of this study are available within the paper and its supplementary information.
