# Supplementary material for: Novel utilization of the seaweed-based deoxy sugars rhamnose and fucose by engineered Corynebacterium glutamicum
Source: Microb Cell Fact. 2026 Jun 11;25:170. doi: 10.1186/s12934-026-03035-5 (PMC13479540; doi:10.1186/s12934-026-03035-5)
Supplement: Supplementary file 1 [file 12934_2026_3035_MOESM1_ESM.docx]

Supplementary information

Novel Utilization of the Seaweed-Based Deoxy Sugars l-Rhamnose and l-Fucose by Engineered *Corynebacterium glutamicum*

**Ida Marie Stephansen^1^, Luciana Fernandes Brito^1^, Oliver Saur Auran^1,^**^+^, **Synne Sæther Lande^1,^**^+^**, Fernando Pérez-García^1,*^**

^1^Department of Biotechnology and Food Science, Faculty of Natural Sciences, NTNU, Trondheim, Norway

^+^Equal contribution

*** Correspondence:** Fernando Pérez-García, [fernando.perez-garcia@ntnu.no](mailto:fernando.perez-garcia@ntnu.no)

| **Table S1.** Oligonucleotide sequences used in this work. | | |
| --- | --- | --- |
| Primer name | Sequence (5’🡪3’) | Description |
| X1Fw | CATCATAACGGTTCTGGC | Forward primer for colony PCR using the plasmid pVWEx1 |
| X1Rv | ATCTTCTCTCATCCGCCA | Reverse primer for colony PCR using the plasmid pVWEx1 |
| ECFw | TTTGCGCCGACATCATAACG | Forward primer for colony PCR using the plasmid pECXT99a |
| ECRv | TACTGCCGCCAGGCAAATTC | Reverse primer for colony PCR using the plasmid pECXT99a |
| pSH1Fw | ATCAACGCCGTTGCCCTTAG | Forward primer for colony PCR using the plasmid pSH1 |
| pSH1Rv | TACTGCCGCCAGGCAAATTC | Reverse primer for colony PCR using the plasmid pSH1 |
| rhaOp-Fw (X1) | *GCATGCCTGCAGGTCGACTCTAGAG***GAAAGGAGGCCCTTCAG**ATGACCTTTCGCAATTGTGTCG | Forward primer for amplification of *rhaBADM* from *E. coli* for the construction of pVWEx1-*rhaBADM,* pVWEx1-*rhaBADM-fucP* and pSH1-*rhaBADM-fucP* |
| rhaOp-Rv (X1) | *AATTCGAGCTCGGTACCCGGGGATC*TTACGGCAGGTAAAACACTTC | Reverse primer for amplification of *rhaBADM* from *E. coli* for the construction of pVWEx1-*rhaBADM* |
| rhaOp-Rv(pSH1) | TTACGGCAGGTAAAACACTTC | Reverse primer for amplification of *rhaBADM* for the construction of pVWEx1-*rhaBADM-fucP* and pSH1-*rhaBADM-fucP* |
| fucIKU-Fw (X1) | *GCCTGCAGGTCGACTCTAGAG***GAAAGGAGGCCCTTCAG**ATGAAAAAAATCAGCTTACCG | Forward primer for amplification of *fucIKU* from *E. coli* for the construction of pVWEx1-*fucIKUA,* pVWEx1-*fucIKUA-iolT2 and* pSH1*-fucIKUA-iolT2* |
| fucIKU-Rv (X1) | TTACGGTGTTACCCCTTTTTTTAAAAG | Reverse primer for amplification of *fucIKU* from *E. coli* for the construction of pVWEx1-*fucIKUA* |
| fucA-Fw (X1) | *AAAAAAAGGGGTAACACCGTAA*ATAGACGCTTCC**GAAAGGAGGCCCTTCAG**ATGGAACGAAATAAACTTGCTCG | Forward primer for amplification of *fucA* from *E. coli* for the construction of pVWEx1-*fucIKUA* |
| fucA-Rv (X1) | *CGAGCTCGGTACCCGGGGATC*TTACTCTTCAATTCGTAACCC | Reverse primer for amplification of *fucA* from *E. coli* for the construction of pVWEx1-*fucIKUA* |
| fucIKUA-Rv (pSH1) | TTACTCTTCAATTCGTAACCC | Reverse primer for amplification of *fucIKUA* for the construction of pVWEx1-*fucIKUA-iolT2* and pSH1*-fucIKUA-iolT2* |
| aldA-Fw (pEC) | *GGAATTCGAGCTCGGTACCCGGG***GAAAGGAGGCCCTTCAG**ATGTCAGTACCCGTTCAACATCC | Forward primer for amplification of *aldA* from *E. coli* into the plasmid pECXT99a |
| aldA-Rv (pEC) | *CCTGCAGGTCGACTCTAGAGGATC*TTAAGACTGTAAATAAACCACC | Reverse primer for amplification of *aldA* from *E. coli* into the plasmid pECXT99a |
| ald-fw (pEC) | *ATGGAATTCGAGCTCGGTACCCGGG***GAAAGGAGGCCCTTCAG**ATGACTGTCTACGCAAATCC | Forward primer for amplification of *ald* from *C. glutamicum* into the plasmid pECXT99a |
| ald-rv (pEC) | *GCCTGCAGGTCGACTCTAGAGGATC*TCAGAACAGTCCGGTTGGG | Reverse primer for amplification of *ald* from *C. glutamicum* into the plasmid pECXT99a |
| rhaT-Fw (pEC) | *ATGGAATTCGAGCTCGGTACCCGGG***GAAAGGAGGCCCTTCAG**ATGAGTAACGCGATTACGAT | Forward primer for amplification of *rhaT* from *E. coli* into the plasmid pECXT99a |
| rhaT-Rv (pEC) | *GCCTGCAGGTCGACTCTAGAGGATC*TTAATTCGCCATGCCGATGC | Reverse primer for amplification of *rhaT* from *E. coli* into the plasmid pECXT99a |
| fucP-Fw (pEC) | *ATGGAATTCGAGCTCGGTACCCGGG***GAAAGGAGGCCCTTCAG**ATGGGAAACACATCAATACAAAC | Forward primer for amplification of *fucP* from *E. coli* into the plasmid pECXT99a |
| fucP-Rv (pEC) | *GCCTGCAGGTCGACTCTAGAGGATC*TCAGTTAGTTGCCGTTTGAG | Reverse primer for amplification of *fucP* from *E. coli* into the plasmid pECXT99a |
| fucP-Fw (pSH1) | *GCAAGAAGTGTTTTACCTGCCGTAA*TTCGAACGCCCC**GAAAGGAGGCCCTTCAG**ATGGGAAACACATCAATACAAACG | Forward primer for amplification of *fucP* for the construction of pVWEx1-*rhaBADM-fucP* and pSH1-*rhaBADM-fucP* |
| fucP-Rv (pSH1) | *AATTCGAGCTCGGTACCCGGGGATC*TCAGTTAGTTGCCGTTTGAGAAC | Reverse primer for amplification of *fucP* for the construction of pVWEx1-*rhaBADM-fucP* and pSH1-*rhaBADM-fucP* |
| iolT2-Fw (pSH1) | *GCAAGAAGTGTTTTACCTGCCGTAA*TTCGAACGCCCC**GAAAGGAGGCCCTTCAG**ATGACGGACATCAAGGCCAC | Forward primer for the amplification of *iolT2* for the construction of pVWEx1-*fucIKUA-iolT2* and pSH1*-fucIKUA-iolT2* |
| iolT2-Rv (pSH1) | *AATTCGAGCTCGGTACCCGGGGATC*TTAAGCCTTCTTGAAGATCTG | Forward primer for the amplification of *iolT2* for the construction of pVWEx1-*fucIKUA-iolT2* and pSH1*-fucIKUA-iolT2* |
| seq_aldA_1 | CCGACATCATAACGGTTCTG | Sequencing primers for *aldA* in pECXT99a-*aldA* |
| seq_aldA_2 | CGTGCGCTTGGTGTGACTAC | Sequencing primers for *aldA* in pECXT99a-*aldA* |
| seq_aldA_3 | ATGCAGGCGGTTCAATTTGG | Sequencing primers for *aldA* in pECXT99a-*aldA* |
| seq_fucIKUA-1 | CATCCGGCTCGTATAATGTG | Sequencing primer for *fucIKUA* in pVWEx1-*fucIKUA* |
| seq_fucIKUA-2 | GTATTAGGGCCGGAAAGTAG | Sequencing primer for *fucIKUA* in pVWEx1-*fucIKUA* |
| seq_fucIKUA-3 | GTACCTGGCTCATCAGTC | Sequencing primer for *fucIKUA* in pVWEx1-*fucIKUA* |
| seq_fucIKUA-4 | GTCGCATGGGTGTTCGTGAG | Sequencing primers for *fucIKUA* in pVWEx1-*fucIKUA* |
| seq_fucIKUA-5 | CCTGGCTGGGAATGAAAGTC | Sequencing primer for *fucIKUA* in pVWEx1-*fucIKUA* |
| seq_fucIKUA-6 | GCCGATGTGCGTACCTACTG | Sequencing primer for *fucIKUA* in pVWEx1-*fucIKUA* |
| seq_fucIKUA-7 | CCGTTTACGGATGTGTACTC | Sequencing primer for *fucIKUA* in pVWEx1-*fucIKUA* |
| seq_fucIKUA-8 | ACTGACTGAATGCCACATCC | Sequencing primer for *fucIKUA* in pVWEx1-*fucIKUA* |
| seq_fucIKUA-9 | ATTTCCGCAGGTCACGATAC | Sequencing primer for *fucIKUA* in pVWEx1-*fucIKUA* |
| seq_fucIKUA-10 | GTTAGTCGGTGGAGGAAGTC | Sequencing primer for *fucIKUA* in pVWEx1-*fucIKUA* |
| seq_fucIKUA-11 | CCCGTGTCCTGACATCATCC | Sequencing primer for *fucIKUA* in pVWEx1-*fucIKUA* |
| seq_fucIKUA-12 | CGGCAGTTTCCATTCTTAACCG | Sequencing primer for *fucIKUA* in pVWEx1-*fucIKUA* |

Italic letters: overlapping sequences for Gibson Assembly. Bold letters: artificial ribosome binding site plus spacer. Regular letters: sequence for PCR amplification.

| **Table S2.** Gene sequences used in this work. | | |
| --- | --- | --- |
| Gene name | Gene sequence (5’->3’) | Description |
| *rhaB* | ATGACCTTTCGCAATTGTGTCGCCGTCGATCTCGGCGCATCCAGTGGGCGCGTGATGCTGGCGCGTTACGAGCGTGAATGCCGCAGCCTGACGCTGCGCGAAATCCATCGTTTTAACAATGGGCTGCATAGTCAGAACGGCTATGTCACCTGGGATGTGGATAGCCTTGAAAGTGCCATTCGCCTTGGATTAAACAAGGTGTGCGAGGAAGGGATTCGTATCGATAGCATTGGGATTGATACCTGGGGCGTGGACTTTGTGCTGCTCGACCAACAGGGTCAGCGTGTGGGCCTGCCCGTTGCTTATCGCGATAGCCGCACCAATGGCCTAATGGCGCAGGCACAACAACAACTCGGCAAACGCGATATTTATCAACGTAGCGGCATCCAGTTTCTGCCCTTCAATACGCTTTATCAGTTG  CGTGCGCTGACGGAGCAACAACCTGAACTTATTCCACACATTGCTCACGCTCTGCTGATGCCGGATTACTTCAGTTATCGCCTGACCGGCAAGATGAACTGGGAATATACCAACGCCACGACCACGCAACTGGTCAATATCAATAGCGACGACTGGGACGAGTCGCTACTGGCGTGGAGCGGGGCCAACAAAGCCTGGTTTGGTCGCCCGACGCATCCGGGTAATGTCATAGGTCACTGGATTTGCCCGCAGGGTAATGAGATTCCAGTGGTCGCCGTTGCCAGCCATGATACCGCCAGCGCGGTTATCGCCTCGCCGTTAAACGGCTCACGTGCTGCTTATCTCTCTTCTGGCACCTGGTCATTGATGGGCTTCGAAAGCCAGACGCCATTTACCAATGACACGGCACTGGCAGCCAACATCACCAATGAAGGCGGGGCGGAAGGTCGCTATCGGGTGCTGAAAAATATTATGGGCTTATGGCTGCTTCAGCGAGTGCTTCAGGAGCAGCAAATCAACGATCTTCCGGCGCTTATCTCCGCGACACAGGCACTTCCGGCTTGCCGCTTCATTATCAATCCCAATGACGATCGCTTTATTAATCCTGAGACGATGTGCAGCGAAATTCAGGCTGCGTGTCGGGAAACGGCGCAACCGATCCCGGAAAGTGATGCTGAACTGGCGCGCTGCATTTTCGACAGTCTGGCGCTGCTGTATGCCGATGTGTTGCATGAGCTGGCGCAGCTGCGCGGTGAAGATTTCTCGCAACTGCATATTGTCGGCGGAGGCTGCCAGAACACGCTGCTCAACCAGCTATGCGCCGATGCCTGCGGTATTCGGGTGATCGCCGGGCCTGTTGAAGCCTCGACGCTCGGCAATATCGGCATCCAGTTAATGACGCTGGATGAACTCAACAATGTGGATGATTTCCGTCAGGTCGTCAGCACCACCGCGAATCTGACCACCTTTACCCCTAATCCTGACAGTGAAATTGCCCACTATGTGGCGCAGATTCACTCTACACGACAGACAAAGGAGCTTTGCGCATGA | Rhamnulosekinase (*E. coli*) NCBI-GeneID: 948399 |
| *rhaA* | ATGACCACTCAACTGGAACAGGCCTGGGAGCTAGCGAAACAGCGTTTCGCGGCGGTGGGGATTGATGTCGAGGAGGCGCTGCGCCAACTTGATCGTTTACCCGTTTCAATGCACTGCTGGCAGGGCGATGATGTTTCCGGTTTTGAAAACCCGGAAGGTTCGCTGACCGGGGGGATTCAGGCCACAGGCAATTATCCGGGCAAAGCGCGTAATGCCAGTGAGCTACGTGCCGATCTGGAACAGGCTATGCGGCTGATTCCGGGGCCGAAACGGCTTAATTTACATGCCATCTATCTGGAATCAGATACGCCAGTCTCGCGCGACCAGATCAAACCAGAGCACTTCAAAAACTGGGTTGAATGGGCGAAAGCCAATCAGCTCGGTCTGGATTTTAACCCCTCCTGCTTTTCGCATCCGCTAAGCGCCGATGGCTTTACGCTTTCCCATGCCGACGACAGCATTCGCCAGTTCTGGATTGATCACTGCAAAGCCAGCCGTCGCGTTTCGGCCTATTTTGGCGAGCAACTCGGCACACCATCGGTGATGAACATCTGGATCCCGGATGGTATGAAAGATATCACCGTTGACCGTCTCGCCCCGCGTCAGCGTCTGCTGGCAGCACTGGATGAGGTGATCAGCGAGAAGCTAAACCCTGCGCACCATATCGACGCCGTTGAGAGCAAATTGTTTGGCATTGGCGCAGAGAGCTACACGGTTGGCTCCAATGAGTTTTACATGGGGTATGCCACCAGCCGCCAGACTGCGCTGTGCCTGGACGCCGGGCACTTCCACCCGACTGAAGTGATTTCCGACAAGATTTCCGCCGCCATGCTGTATGTGCCGCAGTTGCTGCTGCACGTCAGCCGTCCGGTTCGCTGGGACAGCGATCACGTAGTGCTGCTGGATGATGAAACCCAGGCAATTGCCAGTGAGATTGTGCGTCACGATCTGTTTGACCGGGTGCATATCGGCCTTGACTTCTTCGATGCCTCTATCAACCGCATTGCCGCGTGGGTCATTGGTACACGCAATATGAAAAAAGCCCTGCTGCGTGCGTTGCTGGAACCTACCGCTGAGCTGCGCAAGCTGGAAGCGGCGGGCGATTACACTGCGCGTCTGGCACTGCTGGAAGAGCAGAAATCGTTGCCGTGGCAGGCGGTCTGGGAAATGTATTGCCAACGTCACGATACGCCAGCAGGTAGCGAATGGCTGGAGAGCGTGCGGGCTTATGAGAAAGAAATTTTGAGTCGCCGCGGGTAA | L-rhamnose isomerase  (*E. coli)*  NCBI-GeneID: 948400 |
| *rhaD* | ATGCAAAACATTACTCAGTCCTGGTTTGTCCAGGGAATGATCAAAGCCACCACCGACGCCTGGCTGAAAGGCTGGGATGAGCGCAACGGCGGCAACCTGACGCTACGCCTGGATGACGCCGATATCGCACCATATCACGACAATTTCCACCAACAACCGCGCTATATCCCGCTCAGCCAGCCCATGCCTTTACTGGCAAATACACCGTTTATTGTCACCGGCTCGGGCAAATTCTTCCGTAACGTCCAGCTTGATCCTGCGGCTAACTTAGGCATCGTAAAAGTCGACAGCGACGGCGCGGGCTACCACATTCTTTGGGGGTTAACCAACGAAGCCGTCCCCACTTCCGAACTTCCGGCTCACTTCCTTTCCCACTGCGAGCGCATTAAAGCCACCAACGGCAAAGATCGGGTGATCATGCACTGCCACGCCACCAACCTGATCGCCCTCACCTATGTACTTGAAAACGACACCGCGGTCTTCACTCGCCAACTGTGGGAAGGCAGCACCGAGTGTCTGGTGGTATTCCCGGATGGCGTTGGCATTTTGCCGTGGATGGTGCCCGGCACGGACGAAATCGGCCAGGCGACCGCACAAGAGATGCAAAAACATTCGCTGGTGTTGTGGCCCTTCCACGGCGTCTTCGGCAGCGGACCGACGCTGGATGAAACCTTCGGTTTAATCGACACCGCAGAAAAATCAGCACAAGTATTAGTGAAGGTTTATTCGATGGGCGGCATGAAACAGACCATCAGCCGTGAAGAGTTGATAGCGCTCGGCAAGCGTTTCGGCGTTACGCCACTCGCCAGTGCGCTGGCGCTGTAA | Rhamnulose-1-phosphate aldolase  (*E. coli*)  NCBI-GeneID: 948401 |
| *rhaM* | ATGATCCGCAAAGCCTTTGTCATGCAGGTAAACCCCGACGCCCACGAAGAGTATCAGCGTCGGCATAATCCCATCTGGCCAGAACTGGAAGCAGTGCTGAAATCTCACGGTGCGCATAACTACGCCATCTATCTCGACAAAGCGCGTAATCTGCTGTTTGCCATGGTAGAGATTGAATCTGAAGAACGCTGGAATGCGGTTGCCAGCACTGATGTTTGCCAACGTTGGTGGAAATATATGACCGATGTTATGCCCGCTAACCCGGATAACAGCCCGGTGAGTAGCGAGCTGCAAGAAGTGTTTTACCTGCCGTAA | L-rhamnose mutarose  (*E. coli*)  NCBI-GeneID: 948402 |
| *fucI* | ATGAAAAAAATCAGCTTACCGAAAATTGGTATCCGCCCGGTTATTGACGGTCGTCGCATGGGTGTTCGTGAGTCGCTTGAAGAACAAACAATGAATATGGCGAAAGCTACGGCCGCACTGCTGACCGAGAAACTGCGCCATGCCTGCGGAGCTGCCGTCGAGTGTGTCATTTCCGATACCTGTATCGCGGGTATGGCTGAAGCCGCTGCTTGCGAAGAAAAATTCAGCAGTCAGAATGTAGGCCTCACCATTACGGTAACGCCTTGCTGGTGCTATGGCAGTGAAACCATCGACATGGATCCAACCCGCCCGAAGGCCATTTGGGGCTTTAACGGCACTGAACGCCCCGGCGCTGTTTACCTGGCAGCGGCTCTGGCAGCTCACAGCCAGAAAGGCATCCCAGCATTCTCCATTTACGGTCATGACGTTCAGGATGCCGATGACACATCGATTCCTGCCGATGTTGAAGAAAAACTGCTGCGCTTTGCCCGCGCCGGTTTGGCCGTCGCCAGCATGAAAGGTAAAAGCTATCTGTCGCTGGGCGGCGTTTCGATGGGTATCGCCGGTTCCATTGTTGATCACAACTTCTTTGAATCCTGGCTGGGAATGAAAGTCCAGGCGGTGGATATGACCGAACTGCGTCGCCGTATCGATCAGAAGATTTACGACGAAGCCGAATTGGAAATGGCACTGGCCTGGGCTGATAAAAACTTCCGCTATGGCGAAGATGAAAATAACAAACAGTATCAACGTAATGCCGAGCAAAGCCGCGCAGTTCTGCGCGAAAGTTTACTGATGGCGATGTGTATCCGCGACATGATGCAAGGCAACAGCAAACTGGCCGATATTGGTCGCGTGGAAGAATCACTTGGCTACAACGCCATCGCTGCGGGCTTCCAGGGGCAACGTCACTGGACCGATCAATATCCCAATGGTGACACCGCCGAAGCGATCCTCAACAGTTCATTTGACTGGAATGGCGTGCGCGAACCCTTTGTCGTGGCGACCGAAAACGACAGTCTTAACGGCGTGGCAATGCTAATGGGTCACCAGCTCACCGGCACCGCTCAGGTATTTGCCGATGTGCGTACCTACTGGTCACCAGAAGCAATTGAGCGTGTAACGGGGCATAAACTGGATGGACTGGCAGAACACGGCATCATCCATTTGATCAACTCCGGTTCTGCTGCGCTGGACGGTTCCTGTAAACAACGCGACAGCGAAGGTAACCCGACGATGAAGCCACACTGGGAAATCTCTCAGCAAGAGGCTGACGCTTGCCTCGCCGCTACCGAATGGTGCCCGGCGATCCACGAATACTTCCGTGGCGGCGGTTACTCTTCCCGCTTCCTTACCGAAGGCGGCGTCCCGTTCACCATGACTCGTGTCAACATCATCAAAGGCCTGGGACCGGTACTGCAAATCGCGGAAGGCTGGAGCGTGGAATTGCCGAAGGATGTGCATGACATCCTCAACAAACGCACCAACTCAACCTGGCCAACCACCTGGTTTGCACCGCGCCTCACCGGTAAAGGGCCGTTTACGGATGTGTACTCGGTAATGGCGAACTGGGGCGCTAACCATGGGGTTCTGACCATCGGCCACGTTGGCGCAGACTTTATCACTCTCGCCTCCATGCTGCGTATCCCGGTATGTATGCACAACGTTGAAGAGACCAAAGTGTATCGTCCTTCTGCCTGGGCTGCGCACGGCATGGATATTGAAGGCCAGGATTACCGCGCTTGCCAGAACTACGGTCCGTTGTACAAGCGTTAA | L-fucose isomerase  (*E. coli*)  NCBI-GeneID: 946195 |
| *fucK* | ATGTTATCCGGCTATATTGCAGGAGCGATTATGAAACAAGAAGTTATCCTGGTACTCGACTGTGGCGCGACCAATGTCAGGGCCATCGCGGTTAATCGGCAGGGCAAAATTGTTGCCCGCGCCTCAACGCCTAATGCCAGCGATATCGCGATGGAAAACAACACCTGGCACCAGTGGTCTTTAGACGCCATTTTGCAACGCTTTGCTGATTGCTGTCGGCAAATCAATAGTGAACTGACTGAATGCCACATCCGCGGTATCGCCGTCACCACCTTTGGTGTGGATGGCGCTCTGGTAGATAAGCAAGGCAATCTGCTCTATCCGATTATTAGCTGGAAATGTCCGCGAACAGCAGCGGTTATGGACAATATTGAACGGTTAATCTCCGCACAGCGGTTGCAGGCTATTTCTGGCGTCGGAGCCTTTAGTTTCAATACGTTATATAAGTTGGTGTGGTTGAAAGAAAATCATCCACAACTGCTGGAACGCGCGCACGCCTGGCTCTTTATTTCGTCGCTGATTAACCACCGTTTAACCGGCGAATTCACTACTGATATCACGATGGCCGGAACCAGCCAGATGCTGGATATCCAGCAACGCGATTTCAGTCCGCAAATTTTACAAGCCACCGGTATTCCACGCCGACTCTTCCCTCGTCTGGTGGAAGCGGGTGAACAGATTGGTACGCTACAGAACAGCGCCGCAGCAATGCTCGGCTTACCCGTTGGCATACCGGTGATTTCCGCAGGTCACGATACCCAGTTCGCCCTTTTTGGCGCTGGTGCTGAACAAAATGAACCCGTGCTCTCTTCCGGTACATGGGAAATTTTAATGGTTCGCAGCGCCCAGGTTGATACTTCGCTGTTAAGTCAGTACGCCGGTTCCACCTGCGAACTGGATAGCCAGGCAGGGTTGTATAACCCAGGTATGCAATGGCTGGCATCCGGCGTGCTGGAATGGGTGAGAAAACTGTTCTGGACGGCTGAAACACCCTGGCAAATGTTGATTGAAGAAGCTCGTCTGATCGCGCCTGGCGCGGATGGCGTAAAAATGCAGTGTGATTTATTGTCGTGTCAGAACGCTGGCTGGCAAGGAGTGACGCTTAATACCACGCGGGGGCATTTCTATCGCGCGGCGCTGGAAGGGTTAACTGCGCAATTACAGCGCAATCTACAGATGCTGGAAAAAATCGGGCACTTTAAGGCCTCTGAATTATTGTTAGTCGGTGGAGGAAGTCGCAACACATTGTGGAATCAGATTAAAGCCAATATGCTTGATATTCCGGTAAAAGTTCTCGACGACGCCGAAACGACCGTCGCA  GGAGCTGCGCTGTTCGGTTGGTATGGCGTAGGGGAATTTAACAGCCCGGAAGAAGCCCGCGCACAGATTCATTATCAGTACCGTTATTTCTACCCGCAAACTGAACCTGAATTTATAGAGGAAGTGTGA | L-fuculosekinase (*E. coli)*  NCBI-GeneID: 946022 |
| *fucU* | ATGCTGAAAACAATTTCGCCGTTAATTTCTCCCGAACTATTGAAAGTGCTGGCAGAGATGGGACATGGAGATGAAATTATTTTTTCCGATGCTCACTTTCCCGCCCATTCGATGGGACCGCAGGTGATCCGCGCTGATGGCCTGTTGGTGAGCGACTTGCTCCAGGCGATTATCCCGTTATTTGAACTGGACAGTTATGCACCGCCGCTGGTGATGATGGCGGCGGTAGAAGGTGACACTCTCGATCCTGAAGTAGAACGACGTTACCGTAATGCGCTTTCACTACAAGCCCCGTGTCCTGACATCATCCGCATCAATCGTTTTGCGTTTTATGAACGGGCGCAAAAAGCCTTTGCGATCGTTATCACAGGCGAACGAGCGAAGTACGGGAATATTCTTTTAAAAAAAGGGGTAACACCGTAA | L-fucose mutarose  (*E. coli*)  NCBI-GeneID: 945842 |
| *fucA* | ATGGAACGAAATAAACTTGCTCGTCAGATTATTGACACTTGCCTGGAAATGACCCGCCTGGGACTGAACCAGGGGACAGCGGGGAACGTCAGTGTACGTTATCAGGATGGGATGCTGATTACGCCTACAGGCATTCCATATGAAAAACTGACGGAGTCGCATATTGTCTTTATTGATGGCAACGGTAAACATGAGGAAGGAAAGCTCCCCTCAAGCGAATGGCGTTTCCATATGGCAGCCTATCAAAGCAGACCGGATGCCAACGCGGTTGTTCACAATCATGCCGTTCATTGCACGGCAGTTTCCATTCTTAACCGATCGATCCCCGCTATTCACTACATGATTGCGGCGGCTGGCGGTAATTCTATTCCTTGCGCGCCTTATGCGACCTTTGGAACACGCGAACTTTCTGAACATGTTGCGCTGGCTCTCAAAAATCGTAAGGCAACTTTGTTACAACATCATGGGCTTATCGCTTGTGAGGTGAATCTGGAAAAAGCGTTATGGCTGGCGCATGAAGTTGAAGTGCTGGCGCAACTTTACCTGACGACCCTGGCGATTACGGACCCGGTGCCAGTGCTGAGCGATGAAGAGATTGCCGTAGTGCTGGAGAAATTCAAAACCTATGGGTTACGAATTGAAGAGTAA | L-fuculose-phosphate aldolase (*E. coli)*  NCBI-GeneID: 947282 |
| *rhaT* | TTAATTCGCCATGCCGATGCCGACGATGTTAGCGGCGACAATAATCACCACACAACCGAGGCTCAACACCGTTACCGGACGGCGTCCTGCATTGTTCCACTCTTTCAGCACCAGCCCGACGATACCGCCGCACAATACATAGAAACTCATATGCAGCATCCAACTGATGTAGTCATACTGCGCCGGAATGCGGGCGTGGCCCCAGGCATAGAAAAAGAATTGCAGATACCACATCAACCCGCCCAGTGTCGAGAGTAACACATTGTGAATGATCAGCGATTTTGCCAGCGAGAAGTCGGCTTTTAGCGACAAATCCTTCACTTTTGCCAGACGAATAAAACAGAAACCGAGGTTAATGATCGCGCCGCCGCCCATGATGACAACATAGCTTGGCAGAGCGACATACAGTGGATCGACGCC  AAGTGCGGCAGCGGCTTCATGCATCGGTTTTGCGGCGTTCATCGCAAAGGACATCCCGGCAGAGAAAATGCCGCACATCACCGCCAGCACCAGCCCTTTTTTCAGATTGAACTCTTCGGCTTTAATGCCCATCTTGCGCTCTTTCAACTGCCCGGCGCGAGTTACAATCCCTACGCCAATCAGCGCCACCAGAACGCCGAGCAACGTCATGCGTCCGCCTTCGGTGCTAATCAACACATCGAAATTGCCGTTGATAATTGGCGTCATCAGCGTACCGACAATCAACGTAATGCCAATGGCGATGCCAATTCCCATCGACATGCCGAGATAACGCATGGTCAGGCCGTAGTTGATATTACCGATCCCCCACATAGCGCCGAACAGAAAAACAGGCAGTCGCGTAGAGAGACTAAACGAGCTGTAATACGCCCAGAAATTCGGTAGTAACAGGGCGCTGATGGCCCACGGCAGAATAATCCACGAAACAATCCCACCGACTGACCACATGGTTTCCCATGACCATTTTTTTACTTTTTTGAACGGAGCGTAAAAACAGGCTGCACTGGCCGCGCCGATCAAATGCCAAAATATCCCCATCGTAATCGCGTTACTCAT | (*E. coli*)  NCBI-GeneID:  948398 |
| *fucP* | ATGGGAAACACATCAATACAAACGCAGAGTTACCGTGCGGTAGATAAAGATGCAGGGCAAAGCAGAAGTTACATTATTCCATTCGCGCTGCTGTGCTCACTGTTTTTTCTTTGGGCGGTAGCCAATAACCTTAACGACATTTTATTACCTCAATTCCAGCAGGCTTTTACGCTGACAAATTTCCAGGCTGGCCTGATCCAATCGGCCTTTTACTTTGGTTATTTCATTATCCCAATCCCTGCTGGGATATTGATGAAAAAACTCAGTTATAAAGCAGGGATTATTACCGGGTTATTTTTATATGCCTTGGGTGCTGCATTATTCTGGCCCGCCGCAGAAATAATGAACTACACCTTGTTTTTAGTTGGCCTATTTATTATTGCAGCCGGATTAGGTTGTCTGGAAACTGCCGCAAACCCTTTTGTTACGGTATTAGGGCCGGAAAGTAGTGGTCACTTCCGCTTAAATCTTGCGCAAACATTTAACTCGTTTGGCGCAATTATCGCGGTTGTCTTTGGGCAAAGTCTTATTTTGTCTAACGTGCCACATCAATCGCAAGACGTTCTCGATAAAATGTCTCCAGAGCAATTGAGTGCGTATAAACACAGCCTGGTATTATCGGTACAGACACCTTATATGATCATCGTGGCTATCGTGTTACTGGTCGCCCTGCTGATCATGCTGACGAAATTCCCGGCATTGCAGAGTGATAATCACAGTGACGCCAAACAAGGATCGTTCTCCGCATCGCTTTCTCGCCTGGCGCGTATTCGCCACTGGCGCTGGGCGGTATTAGCGCAATTCTGCTATGTCGGCGCACAAACGGCCTGCTGGAGCTATTTGATTCGCTACGCTGTAGAAGAAATTCCAGGTATGACTGCAGGCTTTGCCGCTAACTATTTAACCGGAACCATGGTGTGCTTCTTTATTGGTCGTTTCACCGGTACCTGGCTCATCAGTCGCTTCGCACCACACAAAGTCCTGGCCGCCTACGCATTAATCGCTATGGCACTGTGCCTGATCTCAGCCTTCGCTGGCGGTCATGTGGGCTTAATAGCCCTGACTTTATGCAGCGCCTTTATGTCGATTCAGTACCCAACAATCTTCTCGCTGGGCATTAAGAATCTCGGCCAGGACACCAAATATGGTTCGTCCTTCATCGTTATGACCATTATTGGCGGCGGTATTGTCACTCCGGTCATGGGTTTTGTCAGTGACGCGGCGGGCAACATCCCCACTGCTGAACTGATCCCCGCACTCTGCTTCGCGGTCATCTTTATCTTTGCCCGTTTCCGTTCTCAAACGGCAACTAACTGA | (*E. coli*)  NCBI-GeneID:  947487 |
| *iolT1* | ATGGCTAGTACCTTCATTCAGGCCGACAGCCCTGAAAAAAGTAAGAAGCTGCCCCCACTCACAGAAGGTCCGTATAGAAAGCGGCTATTCTACGTTGCACTAGTTGCGACGTTTGGTGGGCTGCTCTTCGGATATGACACCGGAGTAATCAACGGTGCACTCAACCCAATGACACGTGAGCTCGGACTAACCGCGTTCACCGAGGGTGTTGTAACTTCTTCCCTGCTGTTTGGTGCAGCAGCTGGTGCGATGTTTTTCGGTCGCATTTCCGACAACTGGGGTCGCCGGAAAACAATCATCTCACTTGCAGTAGCTTTCTTTGTCGGCACCATGATCTGCGTGTTTGCTCCATCTTTTGCAGTAATGGTTGTCGGACGTGTGCTTCTTGGACTCGCAGTTGGTGGCGCTTCCACTGTTGTCCCTGTCTACCTGGCTGAACTTGCTCCTTTTGAAAATCCGTGGCTCACTGGCTGGCCGTAATGAGTTGATGATTGTTGTTGGTCAGCTCGCAGCTTTTGTCATCAATGCGATTATTGGAAAATGTTTTTGGACACCACGATGGTGTGTGGCGCTACATGCTGGCAATTGCCGCAATCCCAGCAATTGCCCTCTTCTTTGGAATGCTCCGAGTTCCAGAATCCCCACGCTGGCTTGTTGAGCGAGGACGCATTGATGAGGCTCGCGCAGTTCTTGAAACCATTCGCCCTCTAGAACGTGCCCATGCAGAAGTTGCTGATGTTGAACACCTAGCAAGAGAAGAGCACGCCGTTTCCGAGAAGTCCATGGGCTTAAGGGAAATTTTGTCCAGCAAGTGGCTTGTGCGCATCCTCCTGGTAGGTATCGGATTGGGTGTCGCACAGCAGCTGACCGGCATCAACTCCATCATGTACTACGGCCAGGTTGTTCTCATTGAGGCTGGTTTCTCCGAGAATGCAGCTCTGATCGCCAACGTGGCGCCAGGAGTGATCGCAGTTGTCGGTGCATTCATCGCACTGTGGATGATGGATCGTATCAACCGCCGTACCACCCTCATTACCGGTTATTCTCTCACCACCATTAGCCACGTATTGATCGGTATCGCATCCGTAGCATTCCCAGTCGGCGATCCTCTTCGCCCCTACGTTATCTTGACTCTGGTTGTGGTCTTCGTGGGATCCATGCAGACCTTCCTCAACGTAGCTACCTGGGTTATGCTCTCTGAGCTCTTCCCGCTGGCAATGCGCGGTTTCGCAATCGGTATCTCAGTGTTCTTCCTCTGGATCGCAAACGCGTTCCTCGGATTGTTCTTCCCAACCATCATGGAAGCAGTAGGACTAACCGGAACCTTCTTCATGTTCGCCGGAATCGGTGTGGTTGCCTTGATCTTCATCTACACCCAGGTTCCTGAAACTCGTGGACGTACCTTGGAGGAGATTGATGAGGATGTTATCTTCCGGTGTCATTTTCAACAAGGACATCCGAAAAGGAAAGGTGCACTAA | (*C. glutamicum)*  NCBI-ProteinID: BAB97574 |
| *iolT2* | ATGACGGACATCAAGGCCACATCAAGTACATCGGCCACTACAGCACCAACAGCAGGCCGACCAGCGCGTCGACTTGGACAAATTTCCCTCGTCGCCTGTCTCGGCGGACTTCTCTTCGGCTATGACACCGGTGTCGCCAACGGCGCCGAAGGCCACATGGCACAAGAACTCGGACTCAACGTGCTGCAGCTCGGCGTTGTCATCAGTTCACTGGTTTTCGCTGCAGCCTTTGGCGCGCTGTTCGCTGGGCGTATCTCGGACGAAATCGGGCGTCGAAAAGCAATTATCACTTTGTCCGTGCTGTTCTTCCTCGGATCAATCCTCGTCGTATTCTCCCCCGCCGGTGAGCTGGGGCAGTTCTACGGACCAGGATTTGCCACCTTGGTCACCGGGCGCATCATGTTGGGTCTCGCGGTTGGCGGCGCCTCCACAGTAGTTCCGGTGTACCTCGCTGAACTCGCACCACTAGAAATCCGCGGCTCCCTGACCGGCCGAAACGAGCTTGCTATCGTCACCGGCCAGCTGCTTGCCTTCGTGATCAACGCGCTTATCGCCGTCACCCTACACGGAGTTATTGATGGAATCTGGCGCATCATGTTCGCCGTCTGTGCCCTCCCTGCCGTCGCCCTCTTCCTCGGCATGCTGCGGATGCCGGAATCACCACGCTGGCTGGTCAACCAGGGGCGTTACGACGACGCCCGCCGCGTCATGGAGACCGTCCGTACCCCTGAGCGTGCGAAAGCCGAAATGGATGAAATCATCGCGGTGCACTCTGAAAACAATGCGGCACTTCCTGGTGTTAAGCAGTCTTCGGGCCAGGCTTCAGGCCAGGTTTCTAGCAAGCACACCCACATGTCCATCGGCGAAGTCCTCAGCAACAAATGGCTGGTTCGTCTGCTCATCGCCGGCATCGGTGTTGCAGTTGCCCAGCAGCTCACCGGCATCAACGCCATCATGTACTACGGAACCCGCGTCCTCGAGGAATCCGGCATGAGCGCAGAAATGGCTGTGGTTGCCAACATTGCTTTCGGTGCCGTTGCCGTCATCGGTGGACTGATCGCACTGCGCAACATGGACCGCCTGGATCGCCGCACCACCTTCATCATCGGCCTGTCACTGACCACCACCTTCCACCTTTTGATCGCAGCTGCCGGCACTCTCCTTCCAGAAGGTAACTCCATTCGACCATTCGCCATCATGATCCTTGTTGTTGGGTTCGTGCTCTCCATGCAGACTTTCCTCAACGTTGCAGTGTGGGTGTGGCTGGCGGAAATCTTCCCAGTCCGAATGAAGGGTATCGGCACCGGTATTTCGGTATTCTGCGGTTGGGGCATCAATGGCGTCCTAGCGTTGTTCTTCCCAGCACTGGTCTCCGGCGTGGGTATCACCTTCTCCTTCCTTATCTTCGCAGTCGTCGGAGTCATTGCCCTGGCGTTCGTCACCAAGTTTGTTCCTGAAACCCGTGGCCGCTCACTTGAAGAACTCGATCACGCAGCATTCACCGGCCAGATCTTCAAGAAGGCTTAA | (*C. glutamicum)*  NCBI-ProteinID: BAC00452 |
| *ald* | ATGACTGTCTACGCAAATCCAGGAACCGAAGGCTCGATCGTTAACTATGAAAAGCGCTACGAGAACTACATTGGTGGCAAGTGGGTTCCACCGGTAGAGGGCCAGTACCTTGAGAACATTTCACCTGTCACTGGTGAAGTTTTCTGTGAGGTCGCACGTGGCACCGCAGCGGACGTGGAGCTTGCACTGGATGCTGCACATGCAGCCGCTGATGCGTGGGGCAAGACTTCTGTCGCTGAACGTGCTCTGATCCTGCACCGCATTGCGGACCGCATGGAAGAGCACCTGGAAGAAATCGCAGTTGCAGAAACCTGGGAGAACGGCAAGGCAGTCCGTGAGACTCTTGCTGCAGATATCCCACTGGCAATCGACCACTTCCGCTACTTTGCTGGCGCGATCCGTGCTCAGGAAGATCGTTCCTCACAGATCGACCACAACACTGTTGCTTACCACTTCAACGAGCCAATCGGTGTTGTTGGTCAGATCATTCCTTGGAACTTCCCAATCCTCATGGCTACCTGGAAGCTCGCACCGGCACTTGCTGCAGGTAACGCGATCGTCATGAAGCCAGCTGAGCAGACCCCAGCATCCATTTTGTATCTGATTAACATCATCGGCGATCTCATCCCAGAGGGCGTCCTCAACATCGTCAACGGACTCGGCGGTGAAGCAGGCGCTGCACTGTCCGGCTCTAATCGGATTGGCAAGATTGCTTTCACCGGTTCCACCGAGGTCGGCAAGCTGATCAACCGCGCTGCATCCGACAAGATCATTCCTGTCACCCTGGAGCTCGGCGGTAAGTCCCCATCCATCTTCTTCTCCGATGTTCTGTCACAGGATGACGCCTTCGCAGAGAAGGCAGTTGAAGGCTTCGCGATGTTCGCCCTCAATCAGGGTGAAGTTTGTACCTGTCCTTCCCGTGCACTTGTTCATGAGTCCATCG | Aldehyde dehydrogenase  (*C.* glutamicum) NCBI-ProteinID: CAF20817 |
| *aldA* | ATGTCAGTACCCGTTCAACATCCTATGTATATCGATGGACAGTTTGTTACCTGGCGTGGAGACGCATGGATTGATGTGGTAAACCCTGCTACAGAGGCTGTCATTTCCCGCATACCCGATGGTCAGGCCGAGGATGCCCGTAAGGCAATCGATGCAGCAGAACGTGCACAACCAGAATGGGAAGCGTTGCCTGCTATTGAACGCGCCAGTTGGTTGCGCAAAATCTCCGCCGGGATCCGCGAACGCGCCAGTGAAATCAGTGCGCTGATTGTTGAAGAAGGGGGCAAGATCCAGCAGCTGGCTGAAGTCGAAGTGGCTTTTACTGCCGACTATATCGATTACATGGCGGAGTGGGCACGGCGTTACGAGGGCGAGATTATTCAAAGCGATCGTCCAGGAGAAAATATTCTTTTGTTTAAACGTGCGCTTGGTGTGACTACCGGCATTCTGCCGTGGAACTTCCCGTTCTTCCTCATTGCCCGCAAAATGGCTCCCGCTCTTTTGACCGGTAATACCATCGTCATTAAACCTAGTGAATTTACGCCAAACAATGCGATTGCATTCGCCAAAATCGTCGATGAAATAGGCCTTCCGCGCGGCGTGTTTAACCTTGTACTGGGGCGTGGTGAAACCGTTGGGCAAGAACTGGCGGGTAACCCAAAGGTCGCAATGGTCAGTATGACAGGCAGCGTCTCTGCAGGTGAGAAGATCATGGCGACTGCGGCGAAAAACATCACCAAAGTGTGTCTGGAATTGGGGGGTAAAGCACCAGCTATCGTAATGGACGATGCCGATCTTGAACTGGCAGTCAAAGCCATCGTTGATTCACGCGTCATTAATAGTGGGCAAGTGTGTAACTGTGCAGAACGTGTTTATGTACAGAAAGGCATTTATGATCAGTTCGTCAATCGGCTGGGTGAAGCGATGCAGGCGGTTCAATTTGGTAACCCCGCTGAACGCAACGACATTGCGATGGGGCCGTTGATTAACGCCGCGGCGCTGGAAAGGGTCGAGCAAAAAGTGGCGCGCGCAGTAGAAGAAGGGGCGAGAGTGGCGTTCGGTGGCAAAGCGGTAGAGGGGAAAGGATATTATTATCCGCCGACATTGCTGCTGGATGTTCGCCAGGAAATGTCGATTATGCATGAGGAAACCTTTGGCCCGGTGCTGCCAGTTGTCGCATTTGACACGCTGGAAGATGCTATCTCAATGGCTAATGACAGTGATTACGGCCTGACCTCATCAATCTATACCCAAAATCTGAACGTCGCGATGAAAGCCATTAAAGGGCTGAAGTTTGGTGAAACTTACATCAACCGTGAAAACTTCGAAGCTATGCAAGGCTTCCACGCCGGATGGCGTAAATCCGGTATTGGCGGCGCAGATGGTAAACATGGCTTGCATGAATATCTGCAGACCCAGGTGGTTTATTTACAGTCTTAA | Aldehyde dehydrogenase A (*E. coli)*  NCBI-GeneID: 945672 |
